# Isotypism and phase transitions of (NH_4_)*M*(HSO_4_)(SO_4_)(H_2_O)_2_ (*M* = Fe, Co and Ni) compounds

**DOI:** 10.1107/S2056989025006395

**Published:** 2025-07-23

**Authors:** Matthias Weil

**Affiliations:** a Institute for Chemical Technologies and Analytics, Division of Applied Solid State Chemistry, TU Wien, Getreidemarkt 9/E164-05-1, A-1060 Vienna, Austria; University of Aberdeen, United Kingdom

**Keywords:** crystal structure, isotypism, phase transition, group–subgroup relation, sulfates, hydrogen-bonding

## Abstract

A systematic crystallization study of {(NH_4_)[*M*(HSO_4_)(SO_4_)(H_2_O)_2_]}_*n*_ com­pounds, *catena*-poly[ammonium [[di­aqua­metal(II)]-μ-(hydrogen sulfato)-μ-sul­fato]], revealed that crystals of the *M* = Fe, Co and Ni members could be grown by evaporation of mixed equimolar aqueous solutions of NH_4_HSO_4_ and the corresponding *M*SO_4_ sulfate, whereas for *M* = Mn, Cu, Zn different products were obtained under these conditions. The (NH_4_)*M*(HSO_4_)(SO_4_)(H_2_O)_2_ compounds (*M* = Fe, Co, Ni) show a similar behavior to the magnesium analogue, *i.e.* a reversible structural phase transition from an ordered triclinic crystal structure at *T* = 100 K (*Z* = 2) to a disordered triclinic structure at *T* = 296 K (*Z* = 1). The symmetry relationship between the structure at 296 K and the superstructure at 100 K is of the isomorphic type with index 2. At 100 K, the [*M*O_4_(OH_2_)_2_] octa­hedra are linked by distinct [SO_3_(OH)] and [SO_4_] tetra­hedra into chains. Adjacent chains are linked by very strong hydrogen bonds (O⋯O ≃ 2.5 Å) between the two types of sulfate tetra­hedra into layers. These layers are held together by hydrogen-bonding inter­actions of medium-to-weak strength between the ammonium cations and water mol­ecules. At 296 K, the H atoms of the ammonium tetra­hedron and the H atom between two symmetry-related sulfate groups are disordered. Qu­anti­tative structural comparisons are made between the isotypic (NH_4_)*M*(HSO_4_)(SO_4_)(H_2_O)_2_ structures (*M* = Mg, Fe, Co, Ni) at 296 K and 100 K, respectively.

## Abstract 2

The (NH_4_)*M*(HSO_4_)(SO_4_)(H_2_O)_2_ (*M* = Fe, Co, Ni) compounds are isotypic and exhibit a phase transition from an ordered crystal structure at *T* = 100 K to a disordered crystal structure at *T* = 296 K.

## 1. Chemical context

In an earlier study on (NH_4_)Mg(HSO_4_)(SO_4_)(H_2_O)_2_, it was found that this phase can be crystallized in high yields from equimolar aqueous solutions of NH_4_HSO_4_ and MgSO_4_ by slow evaporation to dryness (Weil & Kolitsch, 2021 ▸). (NH_4_)Mg(HSO_4_)(SO_4_)(H_2_O)_2_ is dimorphic and the corresponding crystal structures were determined and refined on basis of single crystal X-ray data sets recorded at 296 K and 100 K. The crystal structure of the corresponding iron compound (NH_4_)Fe(HSO_4_)(SO_4_)(H_2_O)_2_ was already known previously from a 100 K data set (Heinicke *et al.*, 2004 ▸) and inter­estingly shows isotypism with the crystal structure of the magnesium compound at 296 K.

The aim of the present study is to determine to what extent the corresponding first-row transition-metal compounds (NH_4_)*M*(HSO_4_)(SO_4_)(H_2_O)_2_ (*M*^II^ = Mn, Fe, Co, Ni, Cu, Zn) can be crystallized from aqueous solutions in a similar way to the magnesium compound, and if so, whether they are also dimorphic.

While under these conditions other crystallization products were obtained for *M* = Mn, Cu and Zn, the corresponding (NH_4_)*M*(HSO_4_)(SO_4_)(H_2_O)_2_ compounds could be crystallized for *M* = Fe, Co and Ni, all of which are dimorphic and crystallize isotypically with the corresponding Mg structures at 296 K and 100 K, respectively. The results of these structural investigations are reported in the present article.

## 2. Structural commentary

**Figure 1:**
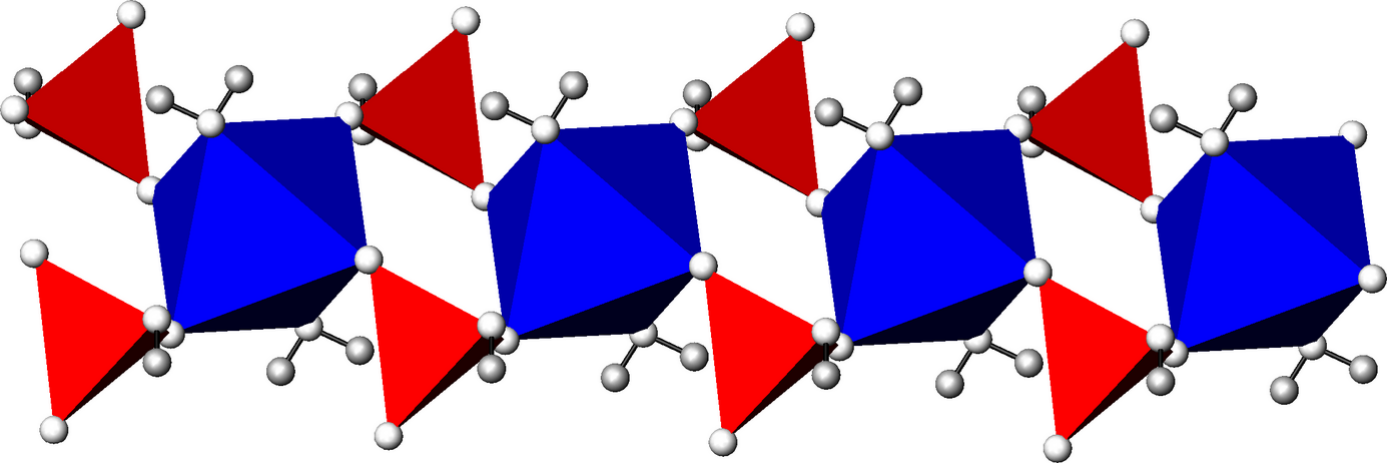
The kröhnkite-type chains in the crystal structures of (NH_4_)*M*(HSO_4_)(SO_4_)(H_2_O)_2_ compounds at 296 K in polyhedral representation. The chains are composed of [*M*O_4_(OH_2_)_2_] octa­hedra (blue) corner-linked by sulfate/hydrogen sulfate tetra­hedra (red).

The title sulfates are new representatives of compounds with kröhnkite-type chains, which are composed of [*M*O_4_(OH_2_)_2_] octa­hedra corner-linked by *X*O_4_ tetra­hedra (Fig. 1 ▸). The widespread occurrence of this motif is related to flexible variations of the octa­hedral-tetra­hedral building units within a chain. Compounds comprising kröhnkite-type chains have been classified into different structure types (Fleck *et al.*, 2002 ▸).

### 2.1. The disordered (NH_4_)*M*(HSO_4_)(SO_4_)(H_2_O)_2_ crystal structure at 296 K

At 296 K, the three isotypic (NH_4_)*M*(HSO_4_)(SO_4_)(H_2_O)_2_ (*M* = Fe, Co, Ni) compounds adopt a triclinic structure (space group *P*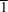, *Z* = 1) corresponding to type E in the classification of compounds with kröhnkite-type chains (Fleck *et al.*, 2002 ▸). As explained below, it is more accurate to describe these compounds at this temperature by the general formula (NH_4_)*M*H(SO_4_)_2_(H_2_O)_2_.

**Figure 2:**
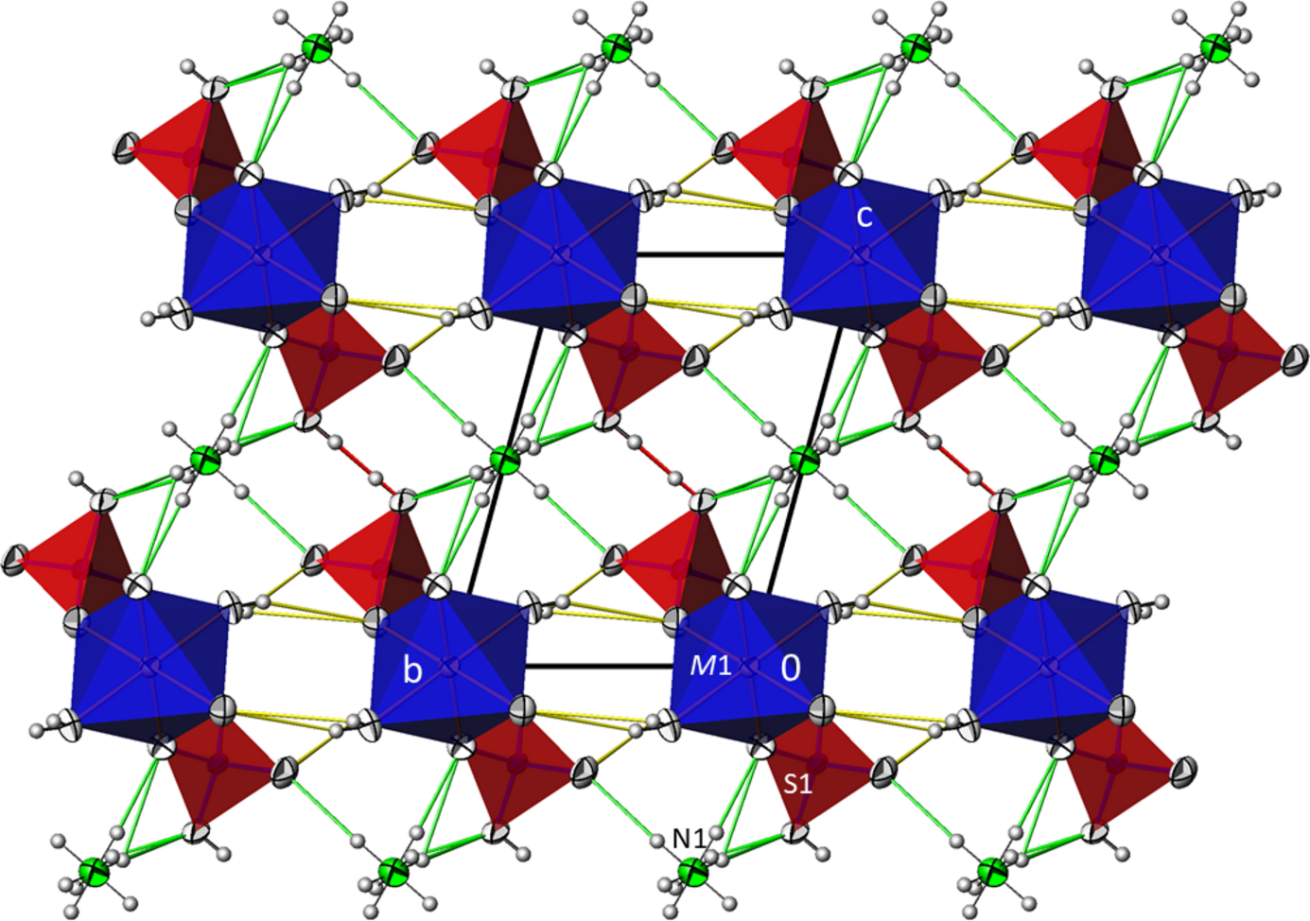
The crystal structure of (NH_4_)*M*(HSO_4_)(SO_4_)(H_2_O)_2_ compounds at 296 K (data from *M* = Co) in a projection along [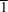
00]. Very strong hydrogen bonds between disordered sulfate/hydrogen sulfate groups are given in red, medium-strong to weak hydrogen bonds involving the water mol­ecules in yellow and those involving the disordered ammonium cations in green. Displacement ellipsoids are drawn at the 74% probability level; H atoms are displayed with arbitrary radius.

The kröhnkite-type chains run along [100] and are joined into sheets parallel to (001) by O—H⋯O hydrogen bonds involving the water mol­ecule (O5) as the donor group. These sheets are held together along [001] through an asymmetric hydrogen bond between two SO_4_ tetra­hedra of two adjacent chains. This hydrogen bond involves the disordered H1*O* atom. The corresponding O1⋯O1^i^ [symmetry code: (i) −*x* − 1, −*y* − 1, −*x* − 1] distance of about 2.48 Å indicates a very strong hydrogen bond (Jeffrey, 1997 ▸). The cohesion within the structure is completed by multiple N—H⋯O hydrogen bonds arising from the ammonium cations, which are located between the sheets (Fig. 2 ▸). The N atom of the ammonium cation is situated at an inversion centre and consequently its hydrogen atoms are equally disordered over two sets of sites. Numerical details of the hydrogen-bonding inter­actions for the 296 K structures are compiled in Tables 1 ▸–3 ▸ ▸ for the three phases.

The [*M*^II^O_4_(OH_2_)_2_] octa­hedra in the 296 K structures exhibit point group symmetry 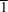 with mean bond lengths of 2.118 Å for *M* = Fe, 2.091 Å for Co, and 2.059 Å for Ni, in good agreement with grand mean values of 2.147 (89), 2.108 (62) and 2.070 (54) Å, respectively, reported in literature (Gagné & Hawthorne, 2020 ▸). The S—O1(H10) bond (average 1.515 Å for the three structures) is the longest in the sulfate tetra­hedron and is about 0.05 Å longer than the S—O bonds to the other O atoms (average 1.462 Å for the three structures). The S—O bond lengths are in good agreement with those given in a review on the sulfate group, for which the grand mean S—O distance is 1.473 Å, with minimum and maximum S—O distances of 1.430 and 1.501 Å, respectively (Hawthorne *et al.*, 2000 ▸).

### 2.2. The ordered (NH_4_)*M*(HSO_4_)(SO_4_)(H_2_O)_2_ crystal structure at 100 K

At 100 K, the three (NH_4_)*M*(HSO_4_)(SO_4_)(H_2_O)_2_ compounds also adopt a triclinic structure (space group *P*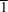, *Z* = 2) corresponding to type E1 (Weil & Kolitsch, 2021 ▸) in the classification of compounds with kröhnkite-type chains. A search in the current version of the Inorganic Crystal Structure Database (ICSD, data release 2024-1; Zagorac *et al.*, 2019 ▸), revealed that, apart from the Mg analogue (Weil & Kolitsch, 2021 ▸) and the title compounds, there are no other members that adopt this structure type.

**Figure 3:**
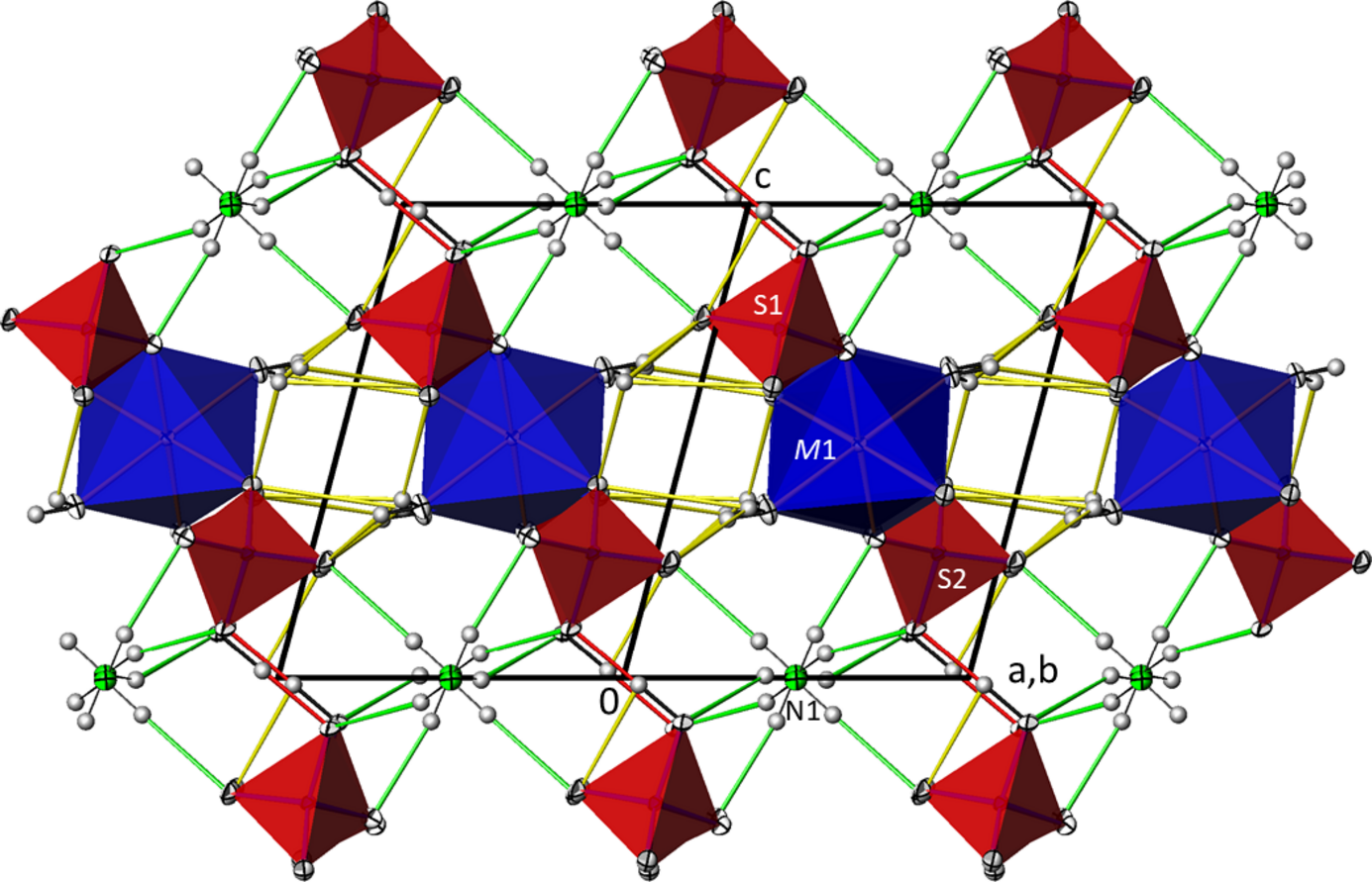
The crystal structure of (NH_4_)*M*(HSO_4_)(SO_4_)(H_2_O)_2_ compounds at 100 K (data from *M* = Co) in a projection along [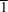
10]. Color code and displacement ellipsoids are as in Fig. 2 ▸.

The crystal structures of the (NH_4_)*M*(HSO_4_)(SO_4_)(H_2_O)_2_ (*M* = Fe, Co, Ni) compounds at 100 K represent a twofold superstructure with ordered hydrogen atoms for the ammonium group and of the hydrogen sulfate group relative to the crystal structure of (NH_4_)*M*H(SO_4_)_2_(H_2_O)_2_ at 296 K. The unit cells of the latter compounds are related to the doubled unit cells of the (NH_4_)*M*(HSO_4_)(SO_4_)(H_2_O)_2_ superstructures at 100 K by the transformation –**a**–**b**, **a**–**b**, **c**. The symmetry relationship (Müller & de la Flor, 2024 ▸) between the substructure at 296 K and the superstructure at 100 K is of isomorphic type with index 2 (i2). All atoms in the superstructure are situated in general positions. [*M*O_4_(OH_2_)] octa­hedra are corner-linked by distinct [SO_3_(OH)] and [SO_4_] tetra­hedra into chains running parallel to [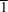
10]. Adjacent chains are joined by O—H⋯O hydrogen bonds between the hydrogen sulfate and sulfate tetra­hedra into sheets extending parallel to (111). The ammonium cations, situated in-between the sheets, and water mol­ecules are also involved in hydrogen-bonding and consolidate the crystal packing (Fig. 3 ▸). In comparison, the bond lengths of all principal building units in the ordered 100 K structure are similar to those in the disordered 296 K structure.

The mean *M*—O bond lengths in the [*M*O_4_(OH_2_)] octa­hedra (2.116 Å for *M* = Fe, 2.087 Å for Co, and 2.053 Å for Ni) hardly differ from those of the 296 K structures. The ordering of the hydrogen atom (H1*O*) between two sulfate tetra­hedra defines distinct S1O_4_ and S2O_3_(OH) groups. The longest bond in the S1O4 tetra­hedron is the bond to O4 (1.491 Å on average for all three structures in contrast to 1.476 Å on average for all other S—O bonds). O4 serves as the acceptor atom for the hydrogen bond with the OH group of the hydrogen sulfate group as donor group. The corresponding [S2O_3_(OH)] tetra­hedron shows the typical distribution of S—O bond lengths in a hydrogen sulfate group, whereby the bond to the OH group (O8) is significantly longer by about 0.09 Å than the remaining three S—O bonds (average for all structures 1.459 Å). The hydrogen bond between the [S2O_3_(OH)] and [S1O_4_] tetra­hedra (O8⋯O4 is on average 2.502 Å) is almost linear [178.6 (12)° for Fe, 177.4 (17)° for Co and 173 (4)° for Ni]. Like the 296 K structures, the other types of O—H⋯O hydrogen-bonding inter­actions are much weaker and involve the water mol­ecules. One of them (O9) shows pairs of bifurcated (*M* = Co, Ni) or trifurcated (*M* = Fe) medium-strong to weak hydrogen bonds. The other water mol­ecule (O10) is involved in one medium-strong and a weak bifurcated hydrogen bond. All H atoms of the ammonium cation are engaged in almost linear hydrogen-bonding inter­actions to the O atoms of the sulfate group as acceptor atoms. Numerical details of hydrogen-bonding inter­actions are compiled in Tables 4 ▸–6 ▸ ▸ for the three 100 K structures.

As already mentioned in the Introduction, the crystal structure of the Fe compound (Heinicke *et al.*, 2004 ▸) was determined in a previous measurement at 100 K in structure type E, *i.e.* in the disordered variant with *Z* = 1, which occurs for all other (NH_4_)*M*(HSO_4_)(SO_4_)(H_2_O)_2_ representatives at 296 K. However, the 100 K data obtained in the present study originate from a slowly cooled (NH_4_)Fe(HSO_4_)(SO_4_)(H_2_O)_2_ crystal and clearly show the ordered variant with *Z* = 2. Whether this difference is possibly due to a different temperature treatment cannot be conclusively clarified, as no details were given in the original publication (Heinicke *et al.*, 2004 ▸). The investigation of the exact ordering temperatures for this and all other (NH_4_)*M*(HSO_4_)(SO_4_)(H_2_O)_2_ representatives, *e.g.* with temperature-dependent powder X-ray diffraction and/or differential scanning calorimetry (DSC) methods, still has to be carried out, but is outside the scope of the present structural study.

## 3. Structural comparison

For a qu­anti­tative structural comparison of the (NH_4_)*M*(HSO_4_)(SO_4_)(H_2_O)_2_ (*M* = Mg, Fe, Co, Ni) structures at 296 K and 100 K, respectively, the program *compstru* (de la Flor *et al.*, 2016 ▸) available at the Bilbao Crystallographic Server (Aroyo *et al.*, 2006 ▸) was used. With *M* = Mg as the reference structure, Table 7 ▸ compiles the maximum distance (*d*_max_) between paired atoms and numerical values regarding the arithmetic mean (*d*_av_) of the distance between paired atoms, the degree of lattice distortion (*δ*), and the measure of similarity (*S*).

As expected for isotypic structures, the comparison between the individual structures (*M* = Fe, Co, Ni) and the reference structure (*M* = Mg) shows very similar numerical values. A clearly recognizable trend cannot be identified, however it may be noted that the numerical parameters for the smallest deviations are always connected with the *M* = Co structure.

## 4. Synthesis and crystallization

Equimolar aqueous solutions of NH_4_HSO_4_ and the corresponding *M*SO_4_ sulfate (*M* = Mn, Fe, Co, Ni, Cu, Zn) were mixed at room temperature and stirred for homogeneity. The mixed solutions were then slowly evaporated to dryness for several days at room temperature. Semi-qu­anti­tative phase analysis of the obtained bulk using the HighScorePlus program (Degen *et al.*, 2014 ▸) revealed the title compounds (NH_4_)*M*(HSO_4_)(SO_4_)(H_2_O)_2_ (*M* = Fe, Co, Ni) as the main products (> 90%_wt_) and [(NH_4_)_2_*M*(SO_4_)_2_(H_2_O)_6_] phases as the minor products. For batches with *M* = Cu and Zn, (NH_4_)_2_*M*(SO_4_)_2_(H_2_O)_6_ phases were the main products and CuSO_4_(H_2_O)_5_ and ZnSO_4_(H_2_O)_6_ the minor products, for both with an approximate phase ratio of 3:1. For the batch with *M* = Mn, langbeinite-type (NH_4_)_2_Mn_2_(SO_4_)_3_ was the only phase obtained.

The (NH_4_)*M*(HSO_4_)(SO_4_)(H_2_O)_2_ single crystals (*M* = Fe, Co, Ni) used for the diffraction studies were broken from larger specimens.

## 5. Refinement

Crystal data, data collection and structure refinement details are summarized in Table 8 ▸. For the low-temperature measurements, the crystals were cooled from 296 K to 100 K within two h. For refinement, coordinates and labeling of atoms of all (NH_4_)*M*(HSO_4_)(SO_4_)(H_2_O)_2_ structures were taken from the isotypic (NH_4_)Mg(HSO_4_)(SO_4_)(H_2_O)_2_ structures for the 296 K and the 100 K data sets (Weil & Kolitsch, 2021 ▸). For all data (296 and 100 K), hydrogen atoms were discernible in difference-Fourier maps and were refined freely. For all 296 K structures, the four ammonium hydrogen atoms (H1*A*–H1*D*) and the H1*O* atom located between two symmetry-related sulfate tetra­hedra are all equally disordered across a centre of symmetry and thus were refined with half-occupancy.

## Supplementary Material

Crystal structure: contains datablock(s) Fe_296K, Co_296K, Ni_296K, Fe_100K, Co_100K, Ni_100K. DOI: 10.1107/S2056989025006395/hb8148sup1.cif

Structure factors: contains datablock(s) Fe_296K. DOI: 10.1107/S2056989025006395/hb8148Fe_296Ksup8.hkl

Structure factors: contains datablock(s) Co_296K. DOI: 10.1107/S2056989025006395/hb8148Co_296Ksup9.hkl

Structure factors: contains datablock(s) Ni_296K. DOI: 10.1107/S2056989025006395/hb8148Ni_296Ksup10.hkl

Structure factors: contains datablock(s) Fe_100K. DOI: 10.1107/S2056989025006395/hb8148Fe_100Ksup11.hkl

Structure factors: contains datablock(s) Co_100K. DOI: 10.1107/S2056989025006395/hb8148Co_100Ksup12.hkl

Structure factors: contains datablock(s) Ni_100K. DOI: 10.1107/S2056989025006395/hb8148Ni_100Ksup13.hkl

CCDC references: 2473608, 2473607, 2473606, 2473605, 2473604, 2473603

Additional supporting information:  crystallographic information; 3D view; checkCIF report

## Figures and Tables

**Table 1:** Hydrogen-bond geometry (Å, °) for *M* = Fe at 296 K

*D*—H⋯*A*	*D*—H	H⋯*A*	*D*⋯*A*	*D*—H⋯*A*
O1—H1*O*⋯O1^i^	0.80 (1)	1.70 (1)	2.4840 (13)	167 (3)
O5—H5*B*⋯O2^ii^	0.79 (2)	2.51 (3)	2.9951 (10)	121 (2)
O5—H5*B*⋯O3^ii^	0.79 (2)	2.52 (2)	3.2141 (11)	148 (2)
N1—H1*A*⋯O3^i^	0.90 (1)	2.02 (1)	2.9218 (7)	175 (6)
N1—H1*B*⋯O1^iii^	0.90 (1)	2.29 (3)	3.1712 (8)	166 (9)
N1—H1*C*⋯O1	0.90 (1)	2.31 (3)	3.1222 (7)	150 (5)
N1—H1*B*⋯O1^iii^	0.90 (1)	2.29 (3)	3.1712 (8)	166 (9)
N1—H1*D*⋯O4^iii^	0.90 (1)	1.98 (1)	2.8736 (7)	174 (7)

Symmetry codes: (i) 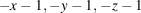; (ii) 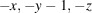; (iii) 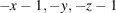.

**Table 2:** Hydrogen-bond geometry (Å, °) for *M* = Co at 296 K

*D*—H⋯*A*	*D*—H	H⋯*A*	*D*⋯*A*	*D*—H⋯*A*
O1—H1*O*⋯O1^i^	0.70 (4)	1.80 (4)	2.4810 (16)	165 (5)
O5—H5*A*⋯O2^ii^	0.74 (3)	2.57 (3)	2.9923 (11)	118 (2)
O5—H5*B*⋯O3^ii^	0.77 (2)	2.51 (2)	3.2020 (13)	149 (2)
N1—H1*A*⋯O3^i^	0.98 (7)	1.96 (7)	2.9298 (8)	169 (7)
N1—H1*D*⋯O1^iii^	0.84 (7)	2.33 (7)	3.1657 (10)	175 (7)
N1—H1*C*⋯O1	0.87 (6)	2.29 (6)	3.1098 (8)	158 (6)
N1—H1*D*⋯O1^iii^	0.84 (7)	2.33 (7)	3.1657 (10)	175 (7)
N1—H1*B*⋯O4^iii^	0.91 (9)	1.97 (9)	2.8773 (7)	176 (8)

Symmetry codes: (i) 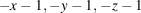; (ii) 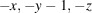; (iii) 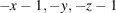.

**Table 3:** Hydrogen-bond geometry (Å, °) for *M* = Ni at 296 K

*D*—H⋯*A*	*D*—H	H⋯*A*	*D*⋯*A*	*D*—H⋯*A*
O1—H1*O*⋯O1^i^	0.85 (1)	1.65 (2)	2.484 (3)	167 (6)
O5—H5*A*⋯O2^ii^	0.84 (1)	2.50 (4)	3.005 (2)	119 (3)
O5—H5*B*⋯O3^ii^	0.84 (1)	2.43 (2)	3.177 (3)	147 (4)
N1—H1*A*⋯O3^i^	0.90 (1)	2.05 (3)	2.9360 (17)	166 (9)
N1—H1*D*⋯O1^iii^	0.90 (1)	2.29 (4)	3.152 (2)	159 (9)
N1—H1*C*⋯O1	0.90 (1)	2.23 (2)	3.0898 (18)	160 (6)
N1—H1*D*⋯O1^iii^	0.90 (1)	2.29 (4)	3.152 (2)	159 (9)
N1—H1*B*⋯O4^iii^	0.90 (1)	2.07 (7)	2.8761 (16)	149 (11)

Symmetry codes: (i) 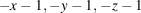; (ii) 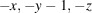; (iii) 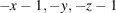.

**Table 4:** Hydrogen-bond geometry (Å, °) for *M* = Fe at 100 K

*D*—H⋯*A*	*D*—H	H⋯*A*	*D*⋯*A*	*D*—H⋯*A*
O8—H1*O*⋯O4^i^	1.040 (16)	1.467 (16)	2.5067 (8)	178.6 (12)
O9—H1*W*⋯O1^ii^	0.816 (17)	2.030 (19)	2.7537 (7)	147.5 (18)
O9—H2*W*⋯O2^iii^	0.875 (17)	2.447 (17)	3.0785 (7)	129.5 (13)
O9—H2*W*⋯O5^iv^	0.875 (17)	2.599 (16)	3.3272 (8)	141.3 (13)
O9—H2*W*⋯O7^iv^	0.875 (17)	2.439 (17)	2.9556 (7)	118.3 (14)
O10—H3*W*⋯O1^v^	0.849 (19)	2.389 (18)	3.1467 (8)	149.0 (15)
O10—H3*W*⋯O2^v^	0.849 (19)	2.541 (19)	3.0541 (7)	119.9 (16)
O10—H4*W*⋯O5^vi^	0.840 (17)	1.946 (18)	2.7115 (7)	151.2 (18)
N1—H3*N*⋯O3^i^	0.873 (12)	1.967 (12)	2.8334 (7)	171.5 (11)
N1—H1*N*⋯O4^iv^	0.871 (12)	2.138 (12)	2.9931 (7)	167.2 (11)
N1—H2*N*⋯O1^iii^	0.857 (13)	2.040 (13)	2.8965 (7)	178.3 (12)
N1—H4*N*⋯O8^vii^	0.890 (14)	2.266 (14)	3.0869 (8)	153.2 (11)

Symmetry codes: (i) 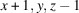; (ii) 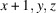; (iii) 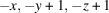; (iv) 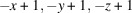; (v) 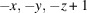; (vi) 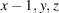; (vii) 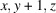.

**Table 5:** Hydrogen-bond geometry (Å, °) for *M* = Co at 100 K

*D*—H⋯*A*	*D*—H	H⋯*A*	*D*⋯*A*	*D*—H⋯*A*
O8—H1*O*⋯O4^i^	0.99 (2)	1.51 (2)	2.5007 (11)	177.4 (17)
O9—H1*W*⋯O1^ii^	0.77 (2)	2.08 (2)	2.7566 (10)	147 (2)
O9—H2*W*⋯O2^iii^	0.82 (2)	2.468 (19)	3.0700 (10)	130.8 (16)
O9—H2*W*⋯O5^iv^	0.82 (2)	2.59 (2)	3.2918 (10)	144.5 (16)
O9—H1*W*⋯O7^iv^	0.77 (2)	2.51 (2)	2.9549 (10)	118.1 (19)
O10—H3*W*⋯O1^v^	0.79 (2)	2.42 (2)	3.1378 (10)	151.0 (18)
O10—H3*W*⋯O2^v^	0.79 (2)	2.58 (2)	3.0492 (10)	119.8 (19)
O10—H4*W*⋯O5^vi^	0.80 (2)	1.98 (2)	2.7127 (10)	152 (2)
N1—H3*N*⋯O3^i^	0.887 (15)	1.955 (15)	2.8328 (10)	170.1 (15)
N1—H1*N*⋯O4^iv^	0.897 (16)	2.114 (16)	2.9890 (11)	164.7 (14)
N1—H2*N*⋯O1^iii^	0.864 (17)	2.038 (17)	2.9017 (11)	178.9 (16)
N1—H4*N*⋯O8^vii^	0.894 (18)	2.252 (18)	3.0777 (11)	153.4 (15)

Symmetry codes: (i) 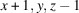; (ii) 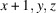; (iii) 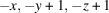; (iv) 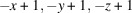; (v) 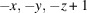; (vi) 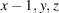; (vii) 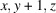.

**Table 6:** Hydrogen-bond geometry (Å, °) for *M* = Ni at 100 K

*D*—H⋯*A*	*D*—H	H⋯*A*	*D*⋯*A*	*D*—H⋯*A*
O8—H1*O*⋯O4^i^	0.88 (4)	1.62 (4)	2.499 (2)	173 (4)
O9—H1*W*⋯O1^ii^	0.80 (5)	2.08 (5)	2.747 (3)	140 (5)
O9—H2*W*⋯O2^iii^	0.65 (4)	2.55 (4)	3.061 (3)	138 (4)
O9—H2*W*⋯O5^iv^	0.65 (4)	2.69 (4)	3.232 (2)	143 (4)
O9—H1*W*⋯O7^iv^	0.80 (5)	2.44 (5)	2.963 (2)	124 (5)
O10—H3*W*⋯O1^v^	0.80 (3)	2.41 (3)	3.115 (2)	148 (3)
O10—H3*W*⋯O2^v^	0.80 (3)	2.59 (4)	3.051 (2)	119 (3)
O10—H4*W*⋯O5^vi^	0.78 (3)	2.00 (3)	2.719 (3)	155 (3)
N1—H3*N*⋯O3^i^	0.87 (3)	1.97 (3)	2.834 (3)	172 (3)
N1—H1*N*⋯O4^iv^	0.93 (3)	2.07 (3)	2.980 (3)	169 (3)
N1—H2*N*⋯O1^iii^	0.91 (3)	2.00 (3)	2.903 (3)	174 (3)
N1—H4*N*⋯O8^vii^	0.79 (6)	2.31 (6)	3.068 (3)	159 (5)

Symmetry codes: (i) 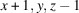; (ii) 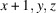; (iii) 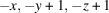; (iv) 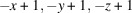; (v) 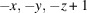; (vi) 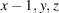; (vii) 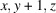.

**Table 7:** Structural comparison of isotypic (NH_4_)*M*(HSO_4_)(SO_4_)(H_2_O)_2_ structures with the *M* = Mg members as references

	Co (100 K)	Fe (100 K)	Ni (100 K)		Co (296 K)	Fe (296 K)	Ni (296 K)
*S*	0.0047	0.0062	0.0074		0.0055	0.0062	0.0078
*d*_max_ (atom)	0.0860 (O1)	0.0893 (O5)	0.1031 (O8)		0.0905 (O3)	0.1003 (O3)	0.0944 (O2)
*d*_av_	0.0469	0.0485	0.0545		0.0521	0.0525	0.0574
*δ*	0.012	0.021	0.017		0.048	0.051	0.062

*S* is the degree of lattice distortion, *d*_max_ (Å) is the maximum difference between two atomic positions, *d*_av_ (Å) is the arithmetic mean of the differences between two atomic positions and *δ* is the measure of similarity.

**Table:** 

	*M* = Fe at 296 K	*M* = Co at 296 K	*M* = Ni at 296 K
Crystal data			
Chemical formula	(NH_4_)[Fe(HSO_4_)(SO_4_)(H_2_O)_2_]	(NH_4_)[Co(HSO_4_)(SO_4_)(H_2_O)_2_]	(NH_4_)[Ni(HSO_4_)(SO_4_)(H_2_O)_2_]
*M*_r_	303.05	306.13	305.91
Crystal system, space group	Triclinic, *P* 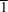	Triclinic, *P* 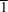	Triclinic, *P* 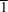
Temperature (K)	296	296	296
*a*, *b*, *c* (Å)	4.6369 (6), 5.8481 (8), 8.4135 (11)	4.6182 (2), 5.8243 (3), 8.3576 (4)	4.5746 (16), 5.7944 (19), 8.347 (2)
α, β, γ (°)	104.010 (2), 98.145 (2), 95.077 (3)	104.2261 (11), 98.1916 (13), 94.7068 (11)	104.049 (11), 98.133 (12), 94.420 (12)
*V* (Å^3^)	217.33 (5)	214.07 (2)	211.04 (12)
*Z*	1	1	1
Radiation type	Mo *K*α	Mo *K*α	Mo *K*α
μ (mm^−1^)	2.26	2.53	2.84
Crystal size (mm)	0.21 × 0.12 × 0.10	0.13 × 0.05 × 0.02	0.12 × 0.06 × 0.01

Data collection			
Diffractometer	Bruker APEXII CCD	Bruker APEXII CCD	Bruker APEXII CCD
Absorption correction	Multi-scan (*SADABS*; Krause *et al.*, 2015 ▸)	Multi-scan (*SADABS*; Krause *et al.*, 2015 ▸)	Multi-scan (*SADABS*; Krause *et al.*, 2015 ▸)
*T*_min_, *T*_max_	0.616, 0.748	0.677, 0.747	0.598, 0.747
No. of measured, independent and observed [*I* > 2σ(*I*)] reflections	8735, 2967, 2634	4115, 1920, 1739	3532, 1591, 1307
*R*_int_	0.028	0.016	0.027
(sin θ/λ)_max_ (Å^−1^)	0.939	0.812	0.769

Refinement			
*R*[*F*^2^ > 2σ(*F*^2^)], *wR*(*F*^2^), *S*	0.022, 0.060, 1.05	0.019, 0.053, 1.05	0.031, 0.077, 1.10
No. of reflections	2967	1920	1591
No. of parameters	96	96	95
No. of restraints	7	0	7
H-atom treatment	All H-atom parameters refined	All H-atom parameters refined	All H-atom parameters refined
Δρ_max_, Δρ_min_ (e Å^−3^)	0.53, −0.45	0.43, −0.40	0.47, −0.59

**Table:** 

	*M* = Fe at 100 K	*M* = Co at 100 K	*M* = Ni at 100 K
Crystal data			
Chemical formula	(NH_4_)[Fe(HSO_4_)(SO_4_)(H_2_O)_2_]	(NH_4_)[Co(HSO_4_)(SO_4_)(H_2_O)_2_]	(NH_4_)[Ni(HSO_4_)(SO_4_)(H_2_O)_2_]
*M*_r_	303.05	306.13	305.91
Crystal system, space group	Triclinic, *P* 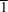	Triclinic, *P* 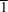	Triclinic, *P* 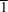
Temperature (K)	100	100	100
*a*, *b*, *c* (Å)	7.0847 (6), 7.7956 (7), 8.3868 (7)	7.0775 (4), 7.7268 (4), 8.3281 (5)	7.0437 (9), 7.6405 (9), 8.3097 (10)
α, β, γ (°)	84.5953 (15), 73.2990 (15), 76.2281 (17)	84.5548 (11), 73.1705 (15), 76.3233 (11)	84.571 (3), 73.376 (3), 76.056 (3)
*V* (Å^3^)	430.74 (6)	423.40 (4)	415.73 (9)
*Z*	2	2	2
Radiation type	Mo *K*α	Mo *K*α	Mo *K*α
μ (mm^−1^)	2.28	2.56	2.88
Crystal size (mm)	0.12 × 0.09 × 0.02	0.12 × 0.09 × 0.02	0.12 × 0.09 × 0.02

Data collection			
Diffractometer	Bruker APEXII CCD	Bruker APEXII CCD	Bruker APEXII CCD
Absorption correction	Multi-scan (*SADABS*; Krause *et al.*, 2015 ▸)	Multi-scan (*SADABS*; Krause *et al.*, 2015 ▸)	Multi-scan (*SADABS*; Krause *et al.*, 2015 ▸)
*T*_min_, *T*_max_	0.619, 0.748	0.669, 0.747	0.609, 0.747
No. of measured, independent and observed [*I* > 2σ(*I*)] reflections	17276, 5854, 5143	8668, 4184, 3507	7657, 3609, 2253
*R*_int_	0.027	0.019	0.031
(sin θ/λ)_max_ (Å^−1^)	0.937	0.854	0.827

Refinement			
*R*[*F*^2^ > 2σ(*F*^2^)], *wR*(*F*^2^), *S*	0.022, 0.062, 1.05	0.023, 0.062, 1.04	0.038, 0.092, 1.02
No. of reflections	5854	4184	3609
No. of parameters	164	164	163
No. of restraints	0	0	0
H-atom treatment	All H-atom parameters refined	All H-atom parameters refined	All H-atom parameters refined
Δρ_max_, Δρ_min_ (e Å^−3^)	0.78, −0.76	0.54, −0.53	0.86, −0.95

Computer programs: *APEX4* and *SAINT* (Bruker, 2020 ▸), *SHELXL* (Sheldrick, 2015 ▸), *ATOMS* (Dowty, 2006 ▸) and *publCIF* (Westrip, 2010 ▸).

## supplementary crystallographic information

### catena-Poly[ammonium [[diaquairon(II)]-µ-(hydrogen sulfato)-µ-sulfato]] (Fe_296K) Crystal data

**Table:**

(NH_4_)[Fe(HSO_4_)(SO_4_)(H_2_O)_2_]	*Z* = 1
*M**_r_* = 303.05	*F*(000) = 154
Triclinic, *P*1	*D*_x_ = 2.315 Mg m^−^^3^
*a* = 4.6369 (6) Å	Mo *K*α radiation, λ = 0.71073 Å
*b* = 5.8481 (8) Å	Cell parameters from 5383 reflections
*c* = 8.4135 (11) Å	θ = 3.6–41.7°
α = 104.010 (2)°	µ = 2.26 mm^−^^1^
β = 98.145 (2)°	*T* = 296 K
γ = 95.077 (3)°	Fragment, light green
*V* = 217.33 (5) Å^3^	0.21 × 0.12 × 0.10 mm

### catena-Poly[ammonium [[diaquairon(II)]-µ-(hydrogen sulfato)-µ-sulfato]] (Fe_296K) Data collection

**Table:**

Bruker APEXII CCD diffractometer	2634 reflections with *I* > 2σ(*I*)
ω– and φ–scans	*R*_int_ = 0.028
Absorption correction: multi-scan (SADABS; Krause *et al.*, 2015)	θ_max_ = 41.9°, θ_min_ = 3.9°
*T*_min_ = 0.616, *T*_max_ = 0.748	*h* = −8→8
8735 measured reflections	*k* = −10→10
2967 independent reflections	*l* = −15→15

### catena-Poly[ammonium [[diaquairon(II)]-µ-(hydrogen sulfato)-µ-sulfato]] (Fe_296K) Refinement

**Table:**

Refinement on *F*^2^	Hydrogen site location: difference Fourier map
Least-squares matrix: full	All H-atom parameters refined
*R*[*F*^2^ > 2σ(*F*^2^)] = 0.022	*w* = 1/[σ^2^(*F*_o_^2^) + (0.0316*P*)^2^ + 0.0223*P*] where *P* = (*F*_o_^2^ + 2*F*_c_^2^)/3
*wR*(*F*^2^) = 0.060	(Δ/σ)_max_ = 0.001
*S* = 1.05	Δρ_max_ = 0.53 e Å^−^^3^
2967 reflections	Δρ_min_ = −0.45 e Å^−^^3^
96 parameters	Extinction correction: SHELXL (Sheldrick, 2015), Fc^*^=kFc[1+0.001xFc^2^λ^3^/sin(2θ)]^-1/4^
7 restraints	Extinction coefficient: 0.216 (10)

### catena-Poly[ammonium [[diaquairon(II)]-µ-(hydrogen sulfato)-µ-sulfato]] (Fe_296K) Special details

**Table:**

Geometry. All esds (except the esd in the dihedral angle between two l.s. planes) are estimated using the full covariance matrix. The cell esds are taken into account individually in the estimation of esds in distances, angles and torsion angles; correlations between esds in cell parameters are only used when they are defined by crystal symmetry. An approximate (isotropic) treatment of cell esds is used for estimating esds involving l.s. planes.

### catena-Poly[ammonium [[diaquairon(II)]-µ-(hydrogen sulfato)-µ-sulfato]] (Fe_296K) Fractional atomic coordinates and isotropic or equivalent isotropic displacement parameters (Å^2^)

**Table:**

	*x*	*y*	*z*	*U*_iso_*/*U*_eq_	Occ. (<1)
Fe1	0.000000	0.000000	0.000000	0.01282 (4)	
S1	−0.59664 (3)	−0.31685 (3)	−0.23953 (2)	0.01243 (4)	
O1	−0.50160 (18)	−0.30338 (11)	−0.40240 (8)	0.02469 (12)	
O2	−0.33634 (14)	−0.28331 (11)	−0.11117 (8)	0.02220 (11)	
O3	−0.76173 (16)	−0.54770 (11)	−0.26110 (8)	0.02589 (13)	
O4	−0.77081 (14)	−0.11909 (11)	−0.19883 (7)	0.02041 (10)	
O5	0.19351 (15)	−0.21979 (12)	0.14262 (9)	0.02261 (11)	
H5A	0.107 (5)	−0.329 (4)	0.156 (3)	0.063 (6)*	
H5B	0.338 (5)	−0.281 (5)	0.131 (3)	0.055 (6)*	
N1	0.000000	0.000000	−0.500000	0.0274 (2)	
H1A	−0.080 (14)	−0.134 (7)	−0.578 (6)	0.093 (18)*	0.5
H1B	−0.129 (15)	0.104 (12)	−0.510 (14)	0.12 (3)*	0.5
H1C	−0.169 (7)	−0.034 (10)	−0.463 (7)	0.071 (14)*	0.5
H1D	−0.078 (14)	0.045 (13)	−0.590 (6)	0.081 (19)*	0.5
H1O	−0.528 (7)	−0.431 (3)	−0.468 (4)	0.038 (9)*	0.5

### catena-Poly[ammonium [[diaquairon(II)]-µ-(hydrogen sulfato)-µ-sulfato]] (Fe_296K) Atomic displacement parameters (Å^2^)

**Table:**

	*U*^11^	*U*^22^	*U*^33^	*U*^12^	*U*^13^	*U*^23^
Fe1	0.01286 (6)	0.01288 (6)	0.01309 (6)	0.00327 (4)	0.00294 (4)	0.00303 (4)
S1	0.01381 (7)	0.01094 (6)	0.01176 (7)	0.00064 (4)	0.00384 (5)	0.00085 (4)
O1	0.0402 (4)	0.0180 (2)	0.0176 (2)	0.0022 (2)	0.0168 (2)	0.00169 (18)
O2	0.0181 (2)	0.0191 (2)	0.0269 (3)	0.00131 (18)	−0.00379 (19)	0.0059 (2)
O3	0.0308 (3)	0.0175 (2)	0.0253 (3)	−0.0097 (2)	0.0077 (2)	0.0006 (2)
O4	0.0220 (2)	0.0238 (2)	0.0188 (2)	0.0119 (2)	0.00839 (19)	0.00588 (19)
O5	0.0210 (3)	0.0206 (2)	0.0311 (3)	0.00657 (19)	0.0057 (2)	0.0139 (2)
N1	0.0354 (6)	0.0237 (4)	0.0217 (4)	0.0013 (4)	−0.0013 (4)	0.0074 (4)

### catena-Poly[ammonium [[diaquairon(II)]-µ-(hydrogen sulfato)-µ-sulfato]] (Fe_296K) Geometric parameters (Å, º)

**Table:**

Fe1—O2	2.1051 (6)	O5—H5A	0.76 (2)
Fe1—O2^i^	2.1051 (6)	O5—H5B	0.79 (2)
Fe1—O4^ii^	2.1174 (6)	N1—H1A	0.901 (10)
Fe1—O4^iii^	2.1174 (6)	N1—H1B	0.900 (10)
Fe1—O5^i^	2.1314 (6)	N1—H1C	0.904 (10)
Fe1—O5	2.1314 (6)	N1—H1D	0.901 (10)
S1—O3	1.4493 (6)	N1—H1A^iv^	0.901 (10)
S1—O2	1.4655 (6)	N1—H1B^iv^	0.900 (10)
S1—O4	1.4694 (6)	N1—H1C^iv^	0.904 (10)
S1—O1	1.5151 (6)	N1—H1D^iv^	0.901 (10)
O1—H1O	0.798 (6)		
			
O2—Fe1—O2^i^	180.0	H1A—N1—H1C	79 (5)
O2—Fe1—O4^ii^	90.82 (3)	H1B—N1—H1C	67 (5)
O2^i^—Fe1—O4^ii^	89.19 (3)	H1A—N1—H1D	73 (5)
O2—Fe1—O4^iii^	89.18 (3)	H1B—N1—H1D	50 (6)
O2^i^—Fe1—O4^iii^	90.81 (3)	H1C—N1—H1D	98 (5)
O4^ii^—Fe1—O4^iii^	180.0	H1A—N1—H1A^iv^	180 (4)
O2—Fe1—O5^i^	91.74 (3)	H1B—N1—H1A^iv^	76 (6)
O2^i^—Fe1—O5^i^	88.26 (3)	H1C—N1—H1A^iv^	101 (5)
O4^ii^—Fe1—O5^i^	93.41 (3)	H1D—N1—H1A^iv^	107 (5)
O4^iii^—Fe1—O5^i^	86.59 (3)	H1A—N1—H1B^iv^	76 (6)
O2—Fe1—O5	88.26 (3)	H1B—N1—H1B^iv^	180.00 (5)
O2^i^—Fe1—O5	91.74 (3)	H1C—N1—H1B^iv^	113 (5)
O4^ii^—Fe1—O5	86.59 (3)	H1D—N1—H1B^iv^	130 (6)
O4^iii^—Fe1—O5	93.41 (3)	H1A^iv^—N1—H1B^iv^	104 (6)
O5^i^—Fe1—O5	180.0	H1A—N1—H1C^iv^	101 (5)
O3—S1—O2	109.74 (4)	H1B—N1—H1C^iv^	113 (5)
O3—S1—O4	113.36 (4)	H1C—N1—H1C^iv^	179.998 (15)
O2—S1—O4	110.13 (4)	H1D—N1—H1C^iv^	82 (5)
O3—S1—O1	109.11 (4)	H1A^iv^—N1—H1C^iv^	79 (5)
O2—S1—O1	109.39 (4)	H1B^iv^—N1—H1C^iv^	67 (5)
O4—S1—O1	104.96 (4)	H1A—N1—H1D^iv^	107 (5)
S1—O1—H1O	111 (3)	H1B—N1—H1D^iv^	130 (6)
S1—O2—Fe1	133.85 (4)	H1C—N1—H1D^iv^	82 (5)
S1—O4—Fe1^v^	132.75 (4)	H1D—N1—H1D^iv^	180 (8)
Fe1—O5—H5A	122.5 (18)	H1A^iv^—N1—H1D^iv^	73 (5)
Fe1—O5—H5B	127.2 (16)	H1B^iv^—N1—H1D^iv^	50 (6)
H5A—O5—H5B	95 (2)	H1C^iv^—N1—H1D^iv^	98 (5)
H1A—N1—H1B	104 (6)		

Symmetry codes: (i) −*x*, −*y*, −*z*; (ii) −*x*−1, −*y*, −*z*; (iii) *x*+1, *y*, *z*; (iv) −*x*, −*y*, −*z*−1; (v) *x*−1, *y*, *z*.

### catena-Poly[ammonium [[diaquairon(II)]-µ-(hydrogen sulfato)-µ-sulfato]] (Fe_296K) Hydrogen-bond geometry (Å, º)

**Table:**

*D*—H···*A*	*D*—H	H···*A*	*D*···*A*	*D*—H···*A*
O1—H1*O*···O1^vi^	0.80 (1)	1.70 (1)	2.4840 (13)	167 (3)
O5—H5*B*···O2^vii^	0.79 (2)	2.51 (3)	2.9951 (10)	121 (2)
O5—H5*B*···O3^vii^	0.79 (2)	2.52 (2)	3.2141 (11)	148 (2)
N1—H1*A*···O3^vi^	0.90 (1)	2.02 (1)	2.9218 (7)	175 (6)
N1—H1*B*···O1^viii^	0.90 (1)	2.29 (3)	3.1712 (8)	166 (9)
N1—H1*C*···O1	0.90 (1)	2.31 (3)	3.1222 (7)	150 (5)
N1—H1*B*···O1^viii^	0.90 (1)	2.29 (3)	3.1712 (8)	166 (9)
N1—H1*D*···O4^viii^	0.90 (1)	1.98 (1)	2.8736 (7)	174 (7)

Symmetry codes: (vi) −*x*−1, −*y*−1, −*z*−1; (vii) −*x*, −*y*−1, −*z*; (viii) −*x*−1, −*y*, −*z*−1.

### catena-Poly[ammonium [[diaquacobalt(II)]-µ-(hydrogen sulfato)-µ-sulfato]] (Co_296K) Crystal data

**Table:**

(NH_4_)[Co(HSO_4_)(SO_4_)(H_2_O)_2_]	*Z* = 1
*M**_r_* = 306.13	*F*(000) = 155
Triclinic, *P*1	*D*_x_ = 2.375 Mg m^−^^3^
*a* = 4.6182 (2) Å	Mo *K*α radiation, λ = 0.71073 Å
*b* = 5.8243 (3) Å	Cell parameters from 2718 reflections
*c* = 8.3576 (4) Å	θ = 2.5–35.3°
α = 104.2261 (11)°	µ = 2.53 mm^−^^1^
β = 98.1916 (13)°	*T* = 296 K
γ = 94.7068 (11)°	Fragment, violet
*V* = 214.07 (2) Å^3^	0.13 × 0.05 × 0.02 mm

### catena-Poly[ammonium [[diaquacobalt(II)]-µ-(hydrogen sulfato)-µ-sulfato]] (Co_296K) Data collection

**Table:**

Bruker APEXII CCD diffractometer	1739 reflections with *I* > 2σ(*I*)
ω– and φ–scans	*R*_int_ = 0.016
Absorption correction: multi-scan (SADABS; Krause *et al.*, 2015)	θ_max_ = 35.3°, θ_min_ = 3.6°
*T*_min_ = 0.677, *T*_max_ = 0.747	*h* = −7→7
4115 measured reflections	*k* = −9→9
1920 independent reflections	*l* = −12→13

### catena-Poly[ammonium [[diaquacobalt(II)]-µ-(hydrogen sulfato)-µ-sulfato]] (Co_296K) Refinement

**Table:**

Refinement on *F*^2^	Hydrogen site location: difference Fourier map
Least-squares matrix: full	All H-atom parameters refined
*R*[*F*^2^ > 2σ(*F*^2^)] = 0.019	*w* = 1/[σ^2^(*F*_o_^2^) + (0.0286*P*)^2^ + 0.0382*P*] where *P* = (*F*_o_^2^ + 2*F*_c_^2^)/3
*wR*(*F*^2^) = 0.053	(Δ/σ)_max_ < 0.001
*S* = 1.05	Δρ_max_ = 0.43 e Å^−^^3^
1920 reflections	Δρ_min_ = −0.39 e Å^−^^3^
96 parameters	Extinction correction: SHELXL (Sheldrick, 2015), Fc^*^=kFc[1+0.001xFc^2^λ^3^/sin(2θ)]^-1/4^
0 restraints	Extinction coefficient: 0.091 (6)

### catena-Poly[ammonium [[diaquacobalt(II)]-µ-(hydrogen sulfato)-µ-sulfato]] (Co_296K) Special details

**Table:**

Geometry. All esds (except the esd in the dihedral angle between two l.s. planes) are estimated using the full covariance matrix. The cell esds are taken into account individually in the estimation of esds in distances, angles and torsion angles; correlations between esds in cell parameters are only used when they are defined by crystal symmetry. An approximate (isotropic) treatment of cell esds is used for estimating esds involving l.s. planes.

### catena-Poly[ammonium [[diaquacobalt(II)]-µ-(hydrogen sulfato)-µ-sulfato]] (Co_296K) Fractional atomic coordinates and isotropic or equivalent isotropic displacement parameters (Å^2^)

**Table:**

	*x*	*y*	*z*	*U*_iso_*/*U*_eq_	Occ. (<1)
Co1	0.000000	0.000000	0.000000	0.01220 (6)	
S1	−0.59237 (5)	−0.31481 (4)	−0.23633 (2)	0.01192 (6)	
O1	−0.4974 (2)	−0.30250 (15)	−0.40060 (10)	0.02333 (16)	
O2	−0.33063 (17)	−0.28375 (14)	−0.10752 (10)	0.02082 (15)	
O3	−0.7600 (2)	−0.54530 (14)	−0.25781 (10)	0.02425 (16)	
O4	−0.76453 (17)	−0.11339 (14)	−0.19483 (9)	0.01918 (14)	
O5	0.19361 (19)	−0.21456 (15)	0.14362 (11)	0.02058 (14)	
H5A	0.108 (6)	−0.320 (4)	0.157 (3)	0.056 (7)*	
H5B	0.338 (5)	−0.271 (5)	0.134 (3)	0.051 (6)*	
N1	0.000000	0.000000	−0.500000	0.0263 (3)	
H1A	−0.103 (16)	−0.149 (12)	−0.576 (9)	0.09 (2)*	0.5
H1B	−0.07 (2)	0.041 (16)	−0.596 (10)	0.09 (3)*	0.5
H1C	−0.155 (13)	−0.046 (11)	−0.462 (8)	0.066 (16)*	0.5
H1D	−0.126 (15)	0.084 (12)	−0.530 (10)	0.064 (18)*	0.5
H1O	−0.528 (9)	−0.416 (7)	−0.456 (5)	0.033 (10)*	0.5

### catena-Poly[ammonium [[diaquacobalt(II)]-µ-(hydrogen sulfato)-µ-sulfato]] (Co_296K) Atomic displacement parameters (Å^2^)

**Table:**

	*U*^11^	*U*^22^	*U*^33^	*U*^12^	*U*^13^	*U*^23^
Co1	0.01195 (8)	0.01226 (8)	0.01283 (8)	0.00288 (5)	0.00301 (5)	0.00313 (6)
S1	0.01354 (10)	0.01038 (9)	0.01119 (9)	0.00054 (7)	0.00395 (7)	0.00082 (7)
O1	0.0382 (4)	0.0170 (3)	0.0162 (3)	0.0016 (3)	0.0156 (3)	0.0011 (3)
O2	0.0171 (3)	0.0176 (3)	0.0252 (3)	0.0004 (2)	−0.0035 (3)	0.0056 (3)
O3	0.0280 (4)	0.0167 (3)	0.0244 (4)	−0.0087 (3)	0.0068 (3)	0.0011 (3)
O4	0.0208 (3)	0.0216 (3)	0.0183 (3)	0.0103 (3)	0.0086 (3)	0.0058 (3)
O5	0.0198 (3)	0.0186 (3)	0.0274 (4)	0.0056 (3)	0.0058 (3)	0.0115 (3)
N1	0.0331 (8)	0.0233 (6)	0.0211 (6)	0.0014 (6)	−0.0008 (5)	0.0070 (5)

### catena-Poly[ammonium [[diaquacobalt(II)]-µ-(hydrogen sulfato)-µ-sulfato]] (Co_296K) Geometric parameters (Å, º)

**Table:**

Co1—O2^i^	2.0821 (8)	O1—H1O	0.70 (4)
Co1—O2	2.0821 (8)	O5—H5A	0.74 (3)
Co1—O4^ii^	2.0890 (7)	O5—H5B	0.77 (2)
Co1—O4^iii^	2.0890 (7)	N1—H1A	0.98 (7)
Co1—O5	2.1016 (8)	N1—H1B	0.91 (9)
Co1—O5^i^	2.1016 (8)	N1—H1C	0.87 (6)
S1—O3	1.4508 (8)	N1—H1D	0.84 (7)
S1—O2	1.4664 (8)	N1—H1B^iv^	0.91 (9)
S1—O4	1.4699 (8)	N1—H1C^iv^	0.87 (6)
S1—O1	1.5155 (8)	N1—H1D^iv^	0.84 (7)
			
O2^i^—Co1—O2	180.0	Co1—O5—H5A	122 (2)
O2^i^—Co1—O4^ii^	90.83 (3)	Co1—O5—H5B	126.8 (18)
O2—Co1—O4^ii^	89.17 (3)	H5A—O5—H5B	97 (3)
O2^i^—Co1—O4^iii^	89.17 (3)	H1A—N1—H1B	76 (6)
O2—Co1—O4^iii^	90.83 (3)	H1A—N1—H1C	70 (5)
O4^ii^—Co1—O4^iii^	180.00 (3)	H1B—N1—H1C	107 (6)
O2^i^—Co1—O5	92.27 (3)	H1A—N1—H1D	94 (5)
O2—Co1—O5	87.73 (3)	H1B—N1—H1D	44 (6)
O4^ii^—Co1—O5	86.33 (3)	H1C—N1—H1D	77 (5)
O4^iii^—Co1—O5	93.67 (3)	H1A—N1—H1B^iv^	104 (6)
O2^i^—Co1—O5^i^	87.73 (3)	H1B—N1—H1B^iv^	180.00 (2)
O2—Co1—O5^i^	92.27 (3)	H1C—N1—H1B^iv^	73 (5)
O4^ii^—Co1—O5^i^	93.67 (3)	H1D—N1—H1B^iv^	136 (6)
O4^iii^—Co1—O5^i^	86.33 (3)	H1A—N1—H1C^iv^	110 (5)
O5—Co1—O5^i^	180.0	H1B—N1—H1C^iv^	73 (5)
O3—S1—O2	109.62 (5)	H1C—N1—H1C^iv^	179.999 (15)
O3—S1—O4	113.51 (5)	H1D—N1—H1C^iv^	103 (5)
O2—S1—O4	110.18 (5)	H1B^iv^—N1—H1C^iv^	107 (6)
O3—S1—O1	109.29 (5)	H1A—N1—H1D^iv^	86 (5)
O2—S1—O1	109.38 (5)	H1B—N1—H1D^iv^	136 (6)
O4—S1—O1	104.71 (5)	H1C—N1—H1D^iv^	103 (5)
S1—O1—H1O	109 (3)	H1D—N1—H1D^iv^	180 (7)
S1—O2—Co1	132.41 (5)	H1B^iv^—N1—H1D^iv^	44 (6)
S1—O4—Co1^v^	132.25 (5)	H1C^iv^—N1—H1D^iv^	77 (5)

Symmetry codes: (i) −*x*, −*y*, −*z*; (ii) −*x*−1, −*y*, −*z*; (iii) *x*+1, *y*, *z*; (iv) −*x*, −*y*, −*z*−1; (v) *x*−1, *y*, *z*.

### catena-Poly[ammonium [[diaquacobalt(II)]-µ-(hydrogen sulfato)-µ-sulfato]] (Co_296K) Hydrogen-bond geometry (Å, º)

**Table:**

*D*—H···*A*	*D*—H	H···*A*	*D*···*A*	*D*—H···*A*
O1—H1*O*···O1^vi^	0.70 (4)	1.80 (4)	2.4810 (16)	165 (5)
O5—H5*A*···O2^vii^	0.74 (3)	2.57 (3)	2.9923 (11)	118 (2)
O5—H5*B*···O3^vii^	0.77 (2)	2.51 (2)	3.2020 (13)	149 (2)
N1—H1*A*···O3^vi^	0.98 (7)	1.96 (7)	2.9298 (8)	169 (7)
N1—H1*D*···O1^viii^	0.84 (7)	2.33 (7)	3.1657 (10)	175 (7)
N1—H1*C*···O1	0.87 (6)	2.29 (6)	3.1098 (8)	158 (6)
N1—H1*D*···O1^viii^	0.84 (7)	2.33 (7)	3.1657 (10)	175 (7)
N1—H1*B*···O4^viii^	0.91 (9)	1.97 (9)	2.8773 (7)	176 (8)

Symmetry codes: (vi) −*x*−1, −*y*−1, −*z*−1; (vii) −*x*, −*y*−1, −*z*; (viii) −*x*−1, −*y*, −*z*−1.

### catena-Poly[ammonium [[diaquanickel(II)]-µ-(hydrogen sulfato)-µ-sulfato]] (Ni_296K) Crystal data

**Table:**

(NH_4_)[Ni(HSO_4_)(SO_4_)(H_2_O)_2_]	*Z* = 1
*M**_r_* = 305.91	*F*(000) = 156
Triclinic, *P*1	*D*_x_ = 2.407 Mg m^−^^3^
*a* = 4.5746 (16) Å	Mo *K*α radiation, λ = 0.71073 Å
*b* = 5.7944 (19) Å	Cell parameters from 1458 reflections
*c* = 8.347 (2) Å	θ = 2.6–32.9°
α = 104.049 (11)°	µ = 2.84 mm^−^^1^
β = 98.133 (12)°	*T* = 296 K
γ = 94.420 (12)°	Plate, green
*V* = 211.04 (12) Å^3^	0.12 × 0.06 × 0.01 mm

### catena-Poly[ammonium [[diaquanickel(II)]-µ-(hydrogen sulfato)-µ-sulfato]] (Ni_296K) Data collection

**Table:**

Bruker APEXII CCD diffractometer	1307 reflections with *I* > 2σ(*I*)
ω– and φ–scans	*R*_int_ = 0.027
Absorption correction: multi-scan (SADABS; Krause *et al.*, 2015)	θ_max_ = 33.2°, θ_min_ = 3.7°
*T*_min_ = 0.598, *T*_max_ = 0.747	*h* = −7→6
3532 measured reflections	*k* = −8→8
1591 independent reflections	*l* = −12→12

### catena-Poly[ammonium [[diaquanickel(II)]-µ-(hydrogen sulfato)-µ-sulfato]] (Ni_296K) Refinement

**Table:**

Refinement on *F*^2^	7 restraints
Least-squares matrix: full	Hydrogen site location: difference Fourier map
*R*[*F*^2^ > 2σ(*F*^2^)] = 0.031	All H-atom parameters refined
*wR*(*F*^2^) = 0.077	*w* = 1/[σ^2^(*F*_o_^2^) + (0.034*P*)^2^ + 0.0522*P*] where *P* = (*F*_o_^2^ + 2*F*_c_^2^)/3
*S* = 1.10	(Δ/σ)_max_ < 0.001
1591 reflections	Δρ_max_ = 0.47 e Å^−^^3^
95 parameters	Δρ_min_ = −0.59 e Å^−^^3^

### catena-Poly[ammonium [[diaquanickel(II)]-µ-(hydrogen sulfato)-µ-sulfato]] (Ni_296K) Special details

**Table:**

Geometry. All esds (except the esd in the dihedral angle between two l.s. planes) are estimated using the full covariance matrix. The cell esds are taken into account individually in the estimation of esds in distances, angles and torsion angles; correlations between esds in cell parameters are only used when they are defined by crystal symmetry. An approximate (isotropic) treatment of cell esds is used for estimating esds involving l.s. planes.

### catena-Poly[ammonium [[diaquanickel(II)]-µ-(hydrogen sulfato)-µ-sulfato]] (Ni_296K) Fractional atomic coordinates and isotropic or equivalent isotropic displacement parameters (Å^2^)

**Table:**

	*x*	*y*	*z*	*U*_iso_*/*U*_eq_	Occ. (<1)
Ni1	0.000000	0.000000	0.000000	0.01194 (10)	
S1	−0.58959 (10)	−0.31438 (8)	−0.23407 (5)	0.01127 (11)	
O1	−0.4942 (4)	−0.3026 (3)	−0.39878 (19)	0.0221 (3)	
O2	−0.3263 (3)	−0.2831 (3)	−0.1046 (2)	0.0189 (3)	
O3	−0.7571 (4)	−0.5462 (3)	−0.2553 (2)	0.0221 (3)	
O4	−0.7659 (3)	−0.1127 (3)	−0.19328 (18)	0.0170 (3)	
O5	0.1932 (4)	−0.2084 (3)	0.1425 (2)	0.0182 (3)	
H5A	0.105 (8)	−0.329 (5)	0.161 (5)	0.057 (12)*	
H5B	0.352 (6)	−0.271 (7)	0.131 (5)	0.068 (14)*	
N1	0.000000	0.000000	−0.500000	0.0247 (6)	
H1A	−0.077 (19)	−0.149 (7)	−0.560 (11)	0.05 (2)*	0.5
H1B	−0.07 (2)	−0.02 (2)	−0.610 (3)	0.07 (3)*	0.5
H1C	−0.167 (8)	−0.054 (11)	−0.469 (8)	0.023 (15)*	0.5
H1D	−0.11 (2)	0.104 (17)	−0.540 (15)	0.09 (4)*	0.5
H1O	−0.475 (14)	−0.439 (5)	−0.459 (6)	0.020 (15)*	0.5

### catena-Poly[ammonium [[diaquanickel(II)]-µ-(hydrogen sulfato)-µ-sulfato]] (Ni_296K) Atomic displacement parameters (Å^2^)

**Table:**

	*U*^11^	*U*^22^	*U*^33^	*U*^12^	*U*^13^	*U*^23^
Ni1	0.01190 (18)	0.01163 (16)	0.01267 (17)	0.00243 (12)	0.00245 (12)	0.00331 (12)
S1	0.0131 (2)	0.00957 (19)	0.0104 (2)	0.00016 (16)	0.00340 (16)	0.00082 (16)
O1	0.0367 (10)	0.0162 (7)	0.0149 (7)	0.0010 (7)	0.0147 (7)	0.0012 (6)
O2	0.0159 (7)	0.0152 (6)	0.0238 (7)	0.0005 (5)	−0.0023 (6)	0.0053 (6)
O3	0.0259 (9)	0.0142 (7)	0.0234 (8)	−0.0080 (6)	0.0058 (6)	0.0017 (6)
O4	0.0180 (7)	0.0195 (7)	0.0165 (6)	0.0090 (6)	0.0074 (6)	0.0057 (6)
O5	0.0179 (8)	0.0162 (7)	0.0238 (7)	0.0041 (6)	0.0042 (6)	0.0101 (6)
N1	0.0324 (17)	0.0203 (13)	0.0194 (13)	0.0000 (12)	−0.0024 (12)	0.0059 (11)

### catena-Poly[ammonium [[diaquanickel(II)]-µ-(hydrogen sulfato)-µ-sulfato]] (Ni_296K) Geometric parameters (Å, º)

**Table:**

Ni1—O5^i^	2.0539 (16)	O5—H5A	0.838 (10)
Ni1—O5	2.0539 (16)	O5—H5B	0.843 (10)
Ni1—O2^i^	2.0592 (16)	N1—H1A	0.901 (10)
Ni1—O2	2.0592 (16)	N1—H1B	0.898 (10)
Ni1—O4^ii^	2.0642 (15)	N1—H1C	0.899 (10)
Ni1—O4^iii^	2.0642 (15)	N1—H1D	0.899 (10)
S1—O3	1.4525 (15)	N1—H1A^iv^	0.901 (10)
S1—O2	1.4678 (16)	N1—H1B^iv^	0.898 (10)
S1—O4	1.4703 (16)	N1—H1C^iv^	0.899 (10)
S1—O1	1.5153 (16)	N1—H1D^iv^	0.899 (10)
O1—H1O	0.845 (10)		
			
O5^i^—Ni1—O5	180.00 (6)	H1A—N1—H1C	66 (6)
O5^i^—Ni1—O2^i^	87.64 (7)	H1B—N1—H1C	99 (7)
O5—Ni1—O2^i^	92.36 (7)	H1A—N1—H1D	108 (7)
O5^i^—Ni1—O2	92.36 (7)	H1B—N1—H1D	58 (8)
O5—Ni1—O2	87.64 (7)	H1C—N1—H1D	87 (7)
O2^i^—Ni1—O2	180.0	H1A—N1—H1A^iv^	179.998 (12)
O5^i^—Ni1—O4^ii^	85.92 (7)	H1B—N1—H1A^iv^	118 (7)
O5—Ni1—O4^ii^	94.08 (7)	H1C—N1—H1A^iv^	114 (6)
O2^i^—Ni1—O4^ii^	89.36 (7)	H1D—N1—H1A^iv^	72 (7)
O2—Ni1—O4^ii^	90.64 (7)	H1A—N1—H1B^iv^	118 (7)
O5^i^—Ni1—O4^iii^	94.08 (7)	H1B—N1—H1B^iv^	180.00 (3)
O5—Ni1—O4^iii^	85.92 (7)	H1C—N1—H1B^iv^	81 (7)
O2^i^—Ni1—O4^iii^	90.64 (7)	H1D—N1—H1B^iv^	122 (8)
O2—Ni1—O4^iii^	89.36 (7)	H1A^iv^—N1—H1B^iv^	62 (7)
O4^ii^—Ni1—O4^iii^	180.00 (11)	H1A—N1—H1C^iv^	114 (6)
O3—S1—O2	109.59 (10)	H1B—N1—H1C^iv^	81 (7)
O3—S1—O4	113.40 (10)	H1C—N1—H1C^iv^	179.999 (12)
O2—S1—O4	110.21 (9)	H1D—N1—H1C^iv^	93 (7)
O3—S1—O1	109.00 (9)	H1A^iv^—N1—H1C^iv^	66 (6)
O2—S1—O1	109.71 (10)	H1B^iv^—N1—H1C^iv^	99 (7)
O4—S1—O1	104.79 (9)	H1A—N1—H1D^iv^	72 (7)
S1—O1—H1O	112 (4)	H1B—N1—H1D^iv^	122 (8)
S1—O2—Ni1	131.92 (10)	H1C—N1—H1D^iv^	93 (7)
S1—O4—Ni1^v^	132.17 (9)	H1D—N1—H1D^iv^	180.00 (6)
Ni1—O5—H5A	124 (3)	H1A^iv^—N1—H1D^iv^	108 (7)
Ni1—O5—H5B	126 (3)	H1B^iv^—N1—H1D^iv^	58 (8)
H5A—O5—H5B	94 (4)	H1C^iv^—N1—H1D^iv^	87 (7)
H1A—N1—H1B	62 (7)		

Symmetry codes: (i) −*x*, −*y*, −*z*; (ii) *x*+1, *y*, *z*; (iii) −*x*−1, −*y*, −*z*; (iv) −*x*, −*y*, −*z*−1; (v) *x*−1, *y*, *z*.

### catena-Poly[ammonium [[diaquanickel(II)]-µ-(hydrogen sulfato)-µ-sulfato]] (Ni_296K) Hydrogen-bond geometry (Å, º)

**Table:**

*D*—H···*A*	*D*—H	H···*A*	*D*···*A*	*D*—H···*A*
O1—H1*O*···O1^vi^	0.85 (1)	1.65 (2)	2.484 (3)	167 (6)
O5—H5*A*···O2^vii^	0.84 (1)	2.50 (4)	3.005 (2)	119 (3)
O5—H5*B*···O3^vii^	0.84 (1)	2.43 (2)	3.177 (3)	147 (4)
N1—H1*A*···O3^vi^	0.90 (1)	2.05 (3)	2.9360 (17)	166 (9)
N1—H1*D*···O1^viii^	0.90 (1)	2.29 (4)	3.152 (2)	159 (9)
N1—H1*C*···O1	0.90 (1)	2.23 (2)	3.0898 (18)	160 (6)
N1—H1*D*···O1^viii^	0.90 (1)	2.29 (4)	3.152 (2)	159 (9)
N1—H1*B*···O4^viii^	0.90 (1)	2.07 (7)	2.8761 (16)	149 (11)

Symmetry codes: (vi) −*x*−1, −*y*−1, −*z*−1; (vii) −*x*, −*y*−1, −*z*; (viii) −*x*−1, −*y*, −*z*−1.

### catena-Poly[ammonium [[diaquairon(II)]-µ-(hydrogen sulfato)-µ-sulfato]] (Fe_100K) Crystal data

**Table:**

(NH_4_)[Fe(HSO_4_)(SO_4_)(H_2_O)_2_]	*Z* = 2
*M**_r_* = 303.05	*F*(000) = 308
Triclinic, *P*1	*D*_x_ = 2.337 Mg m^−^^3^
*a* = 7.0847 (6) Å	Mo *K*α radiation, λ = 0.71073 Å
*b* = 7.7956 (7) Å	Cell parameters from 8457 reflections
*c* = 8.3868 (7) Å	θ = 3.6–41.7°
α = 84.5953 (15)°	µ = 2.28 mm^−^^1^
β = 73.2990 (15)°	*T* = 100 K
γ = 76.2281 (17)°	Fragment, light green
*V* = 430.74 (6) Å^3^	0.12 × 0.09 × 0.02 mm

### catena-Poly[ammonium [[diaquairon(II)]-µ-(hydrogen sulfato)-µ-sulfato]] (Fe_100K) Data collection

**Table:**

Bruker APEXII CCD diffractometer	5143 reflections with *I* > 2σ(*I*)
ω– and φ–scans	*R*_int_ = 0.027
Absorption correction: multi-scan (SADABS; Krause *et al.*, 2015)	θ_max_ = 41.8°, θ_min_ = 3.4°
*T*_min_ = 0.619, *T*_max_ = 0.748	*h* = −13→13
17276 measured reflections	*k* = −13→14
5854 independent reflections	*l* = −15→15

### catena-Poly[ammonium [[diaquairon(II)]-µ-(hydrogen sulfato)-µ-sulfato]] (Fe_100K) Refinement

**Table:**

Refinement on *F*^2^	Hydrogen site location: difference Fourier map
Least-squares matrix: full	All H-atom parameters refined
*R*[*F*^2^ > 2σ(*F*^2^)] = 0.022	*w* = 1/[σ^2^(*F*_o_^2^) + (0.0297*P*)^2^ + 0.0364*P*] where *P* = (*F*_o_^2^ + 2*F*_c_^2^)/3
*wR*(*F*^2^) = 0.062	(Δ/σ)_max_ = 0.001
*S* = 1.05	Δρ_max_ = 0.78 e Å^−^^3^
5854 reflections	Δρ_min_ = −0.76 e Å^−^^3^
164 parameters	Extinction correction: SHELXL (Sheldrick, 2015), Fc^*^=kFc[1+0.001xFc^2^λ^3^/sin(2θ)]^-1/4^
0 restraints	Extinction coefficient: 0.0254 (17)

### catena-Poly[ammonium [[diaquairon(II)]-µ-(hydrogen sulfato)-µ-sulfato]] (Fe_100K) Special details

**Table:**

Geometry. All esds (except the esd in the dihedral angle between two l.s. planes) are estimated using the full covariance matrix. The cell esds are taken into account individually in the estimation of esds in distances, angles and torsion angles; correlations between esds in cell parameters are only used when they are defined by crystal symmetry. An approximate (isotropic) treatment of cell esds is used for estimating esds involving l.s. planes.

### catena-Poly[ammonium [[diaquairon(II)]-µ-(hydrogen sulfato)-µ-sulfato]] (Fe_100K) Fractional atomic coordinates and isotropic or equivalent isotropic displacement parameters (Å^2^)

**Table:**

	*x*	*y*	*z*	*U*_iso_*/*U*_eq_	
Fe1	0.25151 (2)	0.25374 (2)	0.49272 (2)	0.00495 (3)	
S1	−0.20455 (2)	0.38437 (2)	0.73962 (2)	0.00486 (3)	
S2	0.71243 (2)	0.10045 (2)	0.26107 (2)	0.00509 (3)	
O1	−0.40545 (7)	0.35213 (7)	0.75846 (6)	0.00970 (8)	
O2	−0.05984 (7)	0.28137 (6)	0.59849 (6)	0.00805 (7)	
O3	−0.20277 (8)	0.57470 (6)	0.70817 (6)	0.00806 (7)	
O4	−0.14330 (7)	0.33145 (7)	0.89603 (6)	0.00878 (8)	
O5	0.91507 (7)	0.12707 (7)	0.23313 (6)	0.01001 (8)	
O6	0.69127 (7)	−0.08135 (6)	0.30682 (6)	0.00790 (7)	
O7	0.56594 (7)	0.22548 (7)	0.38158 (6)	0.00866 (7)	
O8	0.65788 (8)	0.13443 (7)	0.09282 (6)	0.00920 (8)	
O9	0.26255 (8)	0.46053 (7)	0.63591 (6)	0.00918 (8)	
O10	0.24415 (8)	0.04282 (7)	0.35229 (6)	0.00923 (8)	
N1	0.75683 (8)	0.74104 (7)	0.00607 (6)	0.00962 (9)	
H1W	0.366 (3)	0.468 (3)	0.656 (2)	0.040 (5)*	
H2W	0.219 (3)	0.573 (2)	0.6169 (19)	0.034 (4)*	
H3W	0.291 (3)	−0.067 (2)	0.362 (2)	0.043 (5)*	
H4W	0.136 (3)	0.036 (2)	0.334 (2)	0.036 (4)*	
H1O	0.741 (2)	0.217 (2)	0.0126 (16)	0.060 (6)*	
H1N	0.8604 (18)	0.7089 (16)	0.0468 (14)	0.019 (3)*	
H2N	0.6513 (19)	0.7147 (17)	0.0744 (15)	0.023 (3)*	
H3N	0.7821 (18)	0.6913 (16)	−0.0890 (15)	0.020 (3)*	
H4N	0.7343 (19)	0.8583 (19)	−0.0057 (16)	0.027 (3)*	

### catena-Poly[ammonium [[diaquairon(II)]-µ-(hydrogen sulfato)-µ-sulfato]] (Fe_100K) Atomic displacement parameters (Å^2^)

**Table:**

	*U*^11^	*U*^22^	*U*^33^	*U*^12^	*U*^13^	*U*^23^
Fe1	0.00537 (4)	0.00455 (4)	0.00480 (4)	−0.00135 (3)	−0.00113 (3)	0.00033 (3)
S1	0.00463 (6)	0.00543 (6)	0.00448 (5)	−0.00159 (4)	−0.00111 (4)	0.00087 (4)
S2	0.00486 (6)	0.00560 (6)	0.00485 (5)	−0.00169 (4)	−0.00126 (4)	0.00085 (4)
O1	0.00568 (17)	0.0146 (2)	0.00951 (18)	−0.00435 (15)	−0.00205 (13)	0.00177 (15)
O2	0.00666 (16)	0.00801 (17)	0.00835 (17)	−0.00108 (13)	−0.00005 (13)	−0.00243 (13)
O3	0.01220 (19)	0.00485 (16)	0.00735 (16)	−0.00189 (14)	−0.00339 (14)	0.00101 (13)
O4	0.00992 (18)	0.01136 (19)	0.00653 (16)	−0.00446 (15)	−0.00414 (13)	0.00401 (13)
O5	0.00555 (17)	0.0147 (2)	0.01045 (18)	−0.00411 (15)	−0.00241 (14)	0.00212 (15)
O6	0.01117 (18)	0.00526 (16)	0.00762 (16)	−0.00207 (14)	−0.00348 (14)	0.00165 (13)
O7	0.00748 (17)	0.00758 (17)	0.00980 (17)	−0.00150 (14)	−0.00002 (13)	−0.00261 (13)
O8	0.01154 (19)	0.01164 (19)	0.00667 (16)	−0.00573 (15)	−0.00466 (14)	0.00377 (14)
O9	0.01044 (18)	0.00697 (17)	0.01178 (18)	−0.00233 (14)	−0.00507 (15)	−0.00103 (14)
O10	0.00890 (18)	0.00700 (17)	0.01307 (19)	−0.00151 (14)	−0.00468 (15)	−0.00192 (14)
N1	0.0103 (2)	0.0110 (2)	0.0080 (2)	−0.00397 (18)	−0.00154 (17)	−0.00116 (17)

### catena-Poly[ammonium [[diaquairon(II)]-µ-(hydrogen sulfato)-µ-sulfato]] (Fe_100K) Geometric parameters (Å, º)

**Table:**

Fe1—O2	2.0939 (5)	S2—O7	1.4662 (5)
Fe1—O3^i^	2.1063 (5)	S2—O8	1.5508 (5)
Fe1—O7	2.1192 (5)	O8—H1O	1.040 (16)
Fe1—O6^ii^	2.1222 (5)	O9—H1W	0.816 (17)
Fe1—O9	2.1273 (5)	O9—H2W	0.875 (17)
Fe1—O10	2.1294 (5)	O10—H3W	0.849 (19)
S1—O1	1.4654 (5)	O10—H4W	0.840 (17)
S1—O2	1.4786 (5)	N1—H1N	0.871 (12)
S1—O3	1.4848 (5)	N1—H2N	0.857 (13)
S1—O4	1.4897 (5)	N1—H3N	0.873 (12)
S2—O5	1.4486 (5)	N1—H4N	0.890 (14)
S2—O6	1.4642 (5)		
			
O2—Fe1—O3^i^	91.39 (2)	O5—S2—O7	110.90 (3)
O2—Fe1—O7	179.016 (16)	O6—S2—O7	111.31 (3)
O3^i^—Fe1—O7	87.88 (2)	O5—S2—O8	107.81 (3)
O2—Fe1—O6^ii^	90.08 (2)	O6—S2—O8	103.28 (3)
O3^i^—Fe1—O6^ii^	178.490 (16)	O7—S2—O8	108.40 (3)
O7—Fe1—O6^ii^	90.65 (2)	S1—O2—Fe1	132.45 (3)
O2—Fe1—O9	92.376 (19)	S1—O3—Fe1^i^	131.15 (3)
O3^i^—Fe1—O9	93.37 (2)	S2—O6—Fe1^ii^	132.50 (3)
O7—Fe1—O9	88.326 (19)	S2—O7—Fe1	132.00 (3)
O6^ii^—Fe1—O9	86.24 (2)	S2—O8—H1O	112.0 (7)
O2—Fe1—O10	88.086 (19)	Fe1—O9—H1W	122.3 (13)
O3^i^—Fe1—O10	87.74 (2)	Fe1—O9—H2W	124.8 (10)
O7—Fe1—O10	91.225 (19)	H1W—O9—H2W	97.4 (17)
O6^ii^—Fe1—O10	92.63 (2)	Fe1—O10—H3W	129.4 (11)
O9—Fe1—O10	178.780 (17)	Fe1—O10—H4W	120.2 (12)
O1—S1—O2	108.49 (3)	H3W—O10—H4W	97.2 (17)
O1—S1—O3	111.55 (3)	H1N—N1—H2N	110.8 (12)
O2—S1—O3	109.53 (3)	H1N—N1—H3N	110.5 (12)
O1—S1—O4	110.01 (3)	H2N—N1—H3N	110.0 (12)
O2—S1—O4	110.58 (3)	H1N—N1—H4N	106.1 (12)
O3—S1—O4	106.69 (3)	H2N—N1—H4N	107.8 (13)
O5—S2—O6	114.63 (3)	H3N—N1—H4N	111.5 (13)

Symmetry codes: (i) −*x*, −*y*+1, −*z*+1; (ii) −*x*+1, −*y*, −*z*+1.

### catena-Poly[ammonium [[diaquairon(II)]-µ-(hydrogen sulfato)-µ-sulfato]] (Fe_100K) Hydrogen-bond geometry (Å, º)

**Table:**

*D*—H···*A*	*D*—H	H···*A*	*D*···*A*	*D*—H···*A*
O8—H1*O*···O4^iii^	1.040 (16)	1.467 (16)	2.5067 (8)	178.6 (12)
O9—H1*W*···O1^iv^	0.816 (17)	2.030 (19)	2.7537 (7)	147.5 (18)
O9—H2*W*···O2^i^	0.875 (17)	2.447 (17)	3.0785 (7)	129.5 (13)
O9—H2*W*···O5^v^	0.875 (17)	2.599 (16)	3.3272 (8)	141.3 (13)
O9—H2*W*···O7^v^	0.875 (17)	2.439 (17)	2.9556 (7)	118.3 (14)
O10—H3*W*···O1^vi^	0.849 (19)	2.389 (18)	3.1467 (8)	149.0 (15)
O10—H3*W*···O2^vi^	0.849 (19)	2.541 (19)	3.0541 (7)	119.9 (16)
O10—H4*W*···O5^vii^	0.840 (17)	1.946 (18)	2.7115 (7)	151.2 (18)
N1—H3*N*···O3^iii^	0.873 (12)	1.967 (12)	2.8334 (7)	171.5 (11)
N1—H1*N*···O4^v^	0.871 (12)	2.138 (12)	2.9931 (7)	167.2 (11)
N1—H2*N*···O1^i^	0.857 (13)	2.040 (13)	2.8965 (7)	178.3 (12)
N1—H4*N*···O8^viii^	0.890 (14)	2.266 (14)	3.0869 (8)	153.2 (11)

Symmetry codes: (i) −*x*, −*y*+1, −*z*+1; (iii) *x*+1, *y*, *z*−1; (iv) *x*+1, *y*, *z*; (v) −*x*+1, −*y*+1, −*z*+1; (vi) −*x*, −*y*, −*z*+1; (vii) *x*−1, *y*, *z*; (viii) *x*, *y*+1, *z*.

### catena-Poly[ammonium [[diaquacobalt(II)]-µ-(hydrogen sulfato)-µ-sulfato]] (Co_100K) Crystal data

**Table:**

(NH_4_)[Co(HSO_4_)(SO_4_)(H_2_O)_2_]	*Z* = 2
*M**_r_* = 306.13	*F*(000) = 310
Triclinic, *P*1	*D*_x_ = 2.401 Mg m^−^^3^
*a* = 7.0775 (4) Å	Mo *K*α radiation, λ = 0.71073 Å
*b* = 7.7268 (4) Å	Cell parameters from 3880 reflections
*c* = 8.3281 (5) Å	θ = 2.5–37.9°
α = 84.5548 (11)°	µ = 2.56 mm^−^^1^
β = 73.1705 (15)°	*T* = 100 K
γ = 76.3233 (11)°	Fragment, violet
*V* = 423.40 (4) Å^3^	0.12 × 0.09 × 0.02 mm

### catena-Poly[ammonium [[diaquacobalt(II)]-µ-(hydrogen sulfato)-µ-sulfato]] (Co_100K) Data collection

**Table:**

Bruker APEXII CCD diffractometer	3507 reflections with *I* > 2σ(*I*)
ω– and φ–scans	*R*_int_ = 0.019
Absorption correction: multi-scan (SADABS; Krause *et al.*, 2015)	θ_max_ = 37.4°, θ_min_ = 2.7°
*T*_min_ = 0.669, *T*_max_ = 0.747	*h* = −11→11
8668 measured reflections	*k* = −12→13
4184 independent reflections	*l* = −12→14

### catena-Poly[ammonium [[diaquacobalt(II)]-µ-(hydrogen sulfato)-µ-sulfato]] (Co_100K) Refinement

**Table:**

Refinement on *F*^2^	Hydrogen site location: difference Fourier map
Least-squares matrix: full	All H-atom parameters refined
*R*[*F*^2^ > 2σ(*F*^2^)] = 0.023	*w* = 1/[σ^2^(*F*_o_^2^) + (0.0279*P*)^2^ + 0.0625*P*] where *P* = (*F*_o_^2^ + 2*F*_c_^2^)/3
*wR*(*F*^2^) = 0.062	(Δ/σ)_max_ = 0.001
*S* = 1.04	Δρ_max_ = 0.54 e Å^−^^3^
4184 reflections	Δρ_min_ = −0.53 e Å^−^^3^
164 parameters	Extinction correction: SHELXL (Sheldrick, 2015), Fc^*^=kFc[1+0.001xFc^2^λ^3^/sin(2θ)]^-1/4^
0 restraints	Extinction coefficient: 0.0088 (12)

### catena-Poly[ammonium [[diaquacobalt(II)]-µ-(hydrogen sulfato)-µ-sulfato]] (Co_100K) Special details

**Table:**

Geometry. All esds (except the esd in the dihedral angle between two l.s. planes) are estimated using the full covariance matrix. The cell esds are taken into account individually in the estimation of esds in distances, angles and torsion angles; correlations between esds in cell parameters are only used when they are defined by crystal symmetry. An approximate (isotropic) treatment of cell esds is used for estimating esds involving l.s. planes.

### catena-Poly[ammonium [[diaquacobalt(II)]-µ-(hydrogen sulfato)-µ-sulfato]] (Co_100K) Fractional atomic coordinates and isotropic or equivalent isotropic displacement parameters (Å^2^)

**Table:**

	*x*	*y*	*z*	*U*_iso_*/*U*_eq_	
Co1	0.25150 (2)	0.25333 (2)	0.49323 (2)	0.00461 (4)	
S1	−0.20148 (3)	0.38406 (3)	0.73639 (3)	0.00453 (5)	
S2	0.70903 (3)	0.10225 (3)	0.26461 (3)	0.00474 (5)	
O1	−0.40347 (10)	0.35343 (10)	0.75566 (9)	0.00908 (12)	
O2	−0.05715 (10)	0.27901 (9)	0.59451 (8)	0.00768 (12)	
O3	−0.19659 (11)	0.57524 (9)	0.70390 (8)	0.00763 (12)	
O4	−0.14110 (11)	0.33068 (9)	0.89441 (8)	0.00829 (12)	
O5	0.91201 (10)	0.12872 (10)	0.23703 (9)	0.00941 (12)	
O6	0.68627 (11)	−0.08057 (9)	0.31075 (8)	0.00761 (12)	
O7	0.56238 (10)	0.22842 (9)	0.38606 (9)	0.00817 (12)	
O8	0.65547 (11)	0.13697 (10)	0.09487 (8)	0.00857 (12)	
O9	0.25915 (11)	0.45756 (10)	0.63827 (9)	0.00840 (12)	
O10	0.24613 (11)	0.04547 (10)	0.35051 (9)	0.00849 (12)	
N1	0.75577 (13)	0.74180 (11)	0.00616 (10)	0.00941 (14)	
H1W	0.358 (3)	0.468 (3)	0.655 (3)	0.033 (5)*	
H2W	0.212 (3)	0.564 (3)	0.624 (2)	0.034 (5)*	
H3W	0.291 (3)	−0.058 (3)	0.359 (2)	0.037 (5)*	
H4W	0.142 (3)	0.038 (3)	0.335 (2)	0.031 (5)*	
H1O	0.739 (3)	0.211 (3)	0.015 (2)	0.050 (6)*	
H1N	0.860 (2)	0.708 (2)	0.0516 (19)	0.021 (4)*	
H2N	0.652 (3)	0.713 (2)	0.078 (2)	0.026 (4)*	
H3N	0.786 (2)	0.692 (2)	−0.0922 (19)	0.018 (4)*	
H4N	0.730 (3)	0.861 (2)	−0.004 (2)	0.030 (4)*	

### catena-Poly[ammonium [[diaquacobalt(II)]-µ-(hydrogen sulfato)-µ-sulfato]] (Co_100K) Atomic displacement parameters (Å^2^)

**Table:**

	*U*^11^	*U*^22^	*U*^33^	*U*^12^	*U*^13^	*U*^23^
Co1	0.00482 (6)	0.00409 (6)	0.00483 (6)	−0.00083 (4)	−0.00140 (4)	0.00022 (4)
S1	0.00434 (9)	0.00492 (9)	0.00429 (8)	−0.00129 (7)	−0.00126 (6)	0.00089 (6)
S2	0.00451 (9)	0.00505 (9)	0.00468 (8)	−0.00134 (7)	−0.00135 (6)	0.00081 (6)
O1	0.0053 (3)	0.0131 (3)	0.0095 (3)	−0.0040 (2)	−0.0020 (2)	0.0014 (2)
O2	0.0062 (3)	0.0077 (3)	0.0083 (3)	−0.0009 (2)	−0.0004 (2)	−0.0023 (2)
O3	0.0111 (3)	0.0044 (3)	0.0077 (3)	−0.0014 (2)	−0.0037 (2)	0.0008 (2)
O4	0.0095 (3)	0.0106 (3)	0.0063 (3)	−0.0043 (2)	−0.0041 (2)	0.0041 (2)
O5	0.0049 (3)	0.0135 (3)	0.0102 (3)	−0.0034 (2)	−0.0022 (2)	0.0018 (2)
O6	0.0106 (3)	0.0052 (3)	0.0074 (3)	−0.0019 (2)	−0.0035 (2)	0.0017 (2)
O7	0.0069 (3)	0.0071 (3)	0.0094 (3)	−0.0014 (2)	0.0001 (2)	−0.0025 (2)
O8	0.0106 (3)	0.0114 (3)	0.0059 (3)	−0.0054 (2)	−0.0045 (2)	0.0037 (2)
O9	0.0087 (3)	0.0067 (3)	0.0113 (3)	−0.0017 (2)	−0.0049 (2)	−0.0009 (2)
O10	0.0075 (3)	0.0065 (3)	0.0124 (3)	−0.0008 (2)	−0.0044 (2)	−0.0018 (2)
N1	0.0102 (4)	0.0109 (4)	0.0072 (3)	−0.0036 (3)	−0.0014 (3)	−0.0007 (3)

### catena-Poly[ammonium [[diaquacobalt(II)]-µ-(hydrogen sulfato)-µ-sulfato]] (Co_100K) Geometric parameters (Å, º)

**Table:**

Co1—O2	2.0695 (7)	S2—O7	1.4668 (7)
Co1—O3^i^	2.0771 (7)	S2—O8	1.5506 (7)
Co1—O6^ii^	2.0895 (7)	O8—H1O	0.99 (2)
Co1—O7	2.0905 (7)	O9—H1W	0.77 (2)
Co1—O9	2.0957 (7)	O9—H2W	0.82 (2)
Co1—O10	2.1003 (7)	O10—H3W	0.79 (2)
S1—O1	1.4647 (7)	O10—H4W	0.80 (2)
S1—O2	1.4797 (7)	N1—H1N	0.897 (16)
S1—O3	1.4831 (7)	N1—H2N	0.864 (17)
S1—O4	1.4912 (7)	N1—H3N	0.887 (15)
S2—O5	1.4482 (7)	N1—H4N	0.894 (18)
S2—O6	1.4621 (7)		
			
O2—Co1—O3^i^	89.43 (3)	O5—S2—O7	110.82 (4)
O2—Co1—O6^ii^	91.87 (3)	O6—S2—O7	111.17 (4)
O3^i^—Co1—O6^ii^	178.71 (3)	O5—S2—O8	107.85 (4)
O2—Co1—O7	178.83 (2)	O6—S2—O8	103.30 (4)
O3^i^—Co1—O7	89.54 (3)	O7—S2—O8	108.44 (4)
O6^ii^—Co1—O7	89.16 (3)	S1—O2—Co1	131.08 (4)
O2—Co1—O9	92.65 (3)	S1—O3—Co1^i^	130.79 (4)
O3^i^—Co1—O9	93.92 (3)	S2—O6—Co1^ii^	132.17 (4)
O6^ii^—Co1—O9	85.97 (3)	S2—O7—Co1	130.91 (4)
O7—Co1—O9	87.96 (3)	S2—O8—H1O	112.7 (10)
O2—Co1—O10	87.61 (3)	Co1—O9—H1W	122.4 (15)
O3^i^—Co1—O10	87.02 (3)	Co1—O9—H2W	124.7 (13)
O6^ii^—Co1—O10	93.08 (3)	H1W—O9—H2W	97 (2)
O7—Co1—O10	91.79 (3)	Co1—O10—H3W	129.1 (14)
O9—Co1—O10	179.03 (3)	Co1—O10—H4W	119.8 (14)
O1—S1—O2	108.56 (4)	H3W—O10—H4W	97 (2)
O1—S1—O3	111.65 (4)	H1N—N1—H2N	106.8 (15)
O2—S1—O3	109.44 (4)	H1N—N1—H3N	110.4 (15)
O1—S1—O4	110.01 (4)	H2N—N1—H3N	112.7 (16)
O2—S1—O4	110.63 (4)	H1N—N1—H4N	107.8 (16)
O3—S1—O4	106.55 (4)	H2N—N1—H4N	107.4 (16)
O5—S2—O6	114.78 (4)	H3N—N1—H4N	111.4 (16)

Symmetry codes: (i) −*x*, −*y*+1, −*z*+1; (ii) −*x*+1, −*y*, −*z*+1.

### catena-Poly[ammonium [[diaquacobalt(II)]-µ-(hydrogen sulfato)-µ-sulfato]] (Co_100K) Hydrogen-bond geometry (Å, º)

**Table:**

*D*—H···*A*	*D*—H	H···*A*	*D*···*A*	*D*—H···*A*
O8—H1*O*···O4^iii^	0.99 (2)	1.51 (2)	2.5007 (11)	177.4 (17)
O9—H1*W*···O1^iv^	0.77 (2)	2.08 (2)	2.7566 (10)	147 (2)
O9—H2*W*···O2^i^	0.82 (2)	2.468 (19)	3.0700 (10)	130.8 (16)
O9—H2*W*···O5^v^	0.82 (2)	2.59 (2)	3.2918 (10)	144.5 (16)
O9—H1*W*···O7^v^	0.77 (2)	2.51 (2)	2.9549 (10)	118.1 (19)
O10—H3*W*···O1^vi^	0.79 (2)	2.42 (2)	3.1378 (10)	151.0 (18)
O10—H3*W*···O2^vi^	0.79 (2)	2.58 (2)	3.0492 (10)	119.8 (19)
O10—H4*W*···O5^vii^	0.80 (2)	1.98 (2)	2.7127 (10)	152 (2)
N1—H3*N*···O3^iii^	0.887 (15)	1.955 (15)	2.8328 (10)	170.1 (15)
N1—H1*N*···O4^v^	0.897 (16)	2.114 (16)	2.9890 (11)	164.7 (14)
N1—H2*N*···O1^i^	0.864 (17)	2.038 (17)	2.9017 (11)	178.9 (16)
N1—H4*N*···O8^viii^	0.894 (18)	2.252 (18)	3.0777 (11)	153.4 (15)

Symmetry codes: (i) −*x*, −*y*+1, −*z*+1; (iii) *x*+1, *y*, *z*−1; (iv) *x*+1, *y*, *z*; (v) −*x*+1, −*y*+1, −*z*+1; (vi) −*x*, −*y*, −*z*+1; (vii) *x*−1, *y*, *z*; (viii) *x*, *y*+1, *z*.

### catena-Poly[ammonium [[diaquanickel(II)]-µ-(hydrogen sulfato)-µ-sulfato]] (Ni_100K) Crystal data

**Table:**

(NH_4_)[Ni(HSO_4_)(SO_4_)(H_2_O)_2_]	*Z* = 2
*M**_r_* = 305.91	*F*(000) = 312
Triclinic, *P*1	*D*_x_ = 2.444 Mg m^−^^3^
*a* = 7.0437 (9) Å	Mo *K*α radiation, λ = 0.71073 Å
*b* = 7.6405 (9) Å	Cell parameters from 1834 reflections
*c* = 8.3097 (10) Å	θ = 2.6–35.4°
α = 84.571 (3)°	µ = 2.88 mm^−^^1^
β = 73.376 (3)°	*T* = 100 K
γ = 76.056 (3)°	Plate, green
*V* = 415.73 (9) Å^3^	0.12 × 0.09 × 0.02 mm

### catena-Poly[ammonium [[diaquanickel(II)]-µ-(hydrogen sulfato)-µ-sulfato]] (Ni_100K) Data collection

**Table:**

Bruker APEXII CCD diffractometer	2253 reflections with *I* > 2σ(*I*)
ω– and φ–scans	*R*_int_ = 0.031
Absorption correction: multi-scan (SADABS; Krause *et al.*, 2015)	θ_max_ = 36.0°, θ_min_ = 2.8°
*T*_min_ = 0.609, *T*_max_ = 0.747	*h* = −11→11
7657 measured reflections	*k* = −12→10
3609 independent reflections	*l* = −13→13

### catena-Poly[ammonium [[diaquanickel(II)]-µ-(hydrogen sulfato)-µ-sulfato]] (Ni_100K) Refinement

**Table:**

Refinement on *F*^2^	0 restraints
Least-squares matrix: full	Hydrogen site location: difference Fourier map
*R*[*F*^2^ > 2σ(*F*^2^)] = 0.038	All H-atom parameters refined
*wR*(*F*^2^) = 0.092	*w* = 1/[σ^2^(*F*_o_^2^) + (0.0362*P*)^2^] where *P* = (*F*_o_^2^ + 2*F*_c_^2^)/3
*S* = 1.01	(Δ/σ)_max_ < 0.001
3609 reflections	Δρ_max_ = 0.86 e Å^−^^3^
163 parameters	Δρ_min_ = −0.95 e Å^−^^3^

### catena-Poly[ammonium [[diaquanickel(II)]-µ-(hydrogen sulfato)-µ-sulfato]] (Ni_100K) Special details

**Table:**

Geometry. All esds (except the esd in the dihedral angle between two l.s. planes) are estimated using the full covariance matrix. The cell esds are taken into account individually in the estimation of esds in distances, angles and torsion angles; correlations between esds in cell parameters are only used when they are defined by crystal symmetry. An approximate (isotropic) treatment of cell esds is used for estimating esds involving l.s. planes.

### catena-Poly[ammonium [[diaquanickel(II)]-µ-(hydrogen sulfato)-µ-sulfato]] (Ni_100K) Fractional atomic coordinates and isotropic or equivalent isotropic displacement parameters (Å^2^)

**Table:**

	*x*	*y*	*z*	*U*_iso_*/*U*_eq_	
Ni1	0.25124 (5)	0.25282 (4)	0.49418 (4)	0.00539 (7)	
S1	−0.20008 (8)	0.38376 (7)	0.73454 (6)	0.00486 (11)	
S2	0.70693 (8)	0.10508 (7)	0.26711 (6)	0.00487 (11)	
O1	−0.4025 (3)	0.3526 (2)	0.75282 (19)	0.0092 (3)	
O2	−0.0549 (2)	0.2761 (2)	0.59314 (19)	0.0075 (3)	
O3	−0.1951 (3)	0.5767 (2)	0.70128 (19)	0.0071 (3)	
O4	−0.1400 (3)	0.3306 (2)	0.89363 (19)	0.0083 (3)	
O5	0.9098 (2)	0.1337 (2)	0.24048 (19)	0.0084 (3)	
O6	0.6869 (3)	−0.0809 (2)	0.31174 (19)	0.0083 (3)	
O7	0.5585 (2)	0.2307 (2)	0.39011 (19)	0.0077 (3)	
O8	0.6523 (3)	0.1411 (2)	0.09759 (19)	0.0084 (3)	
O9	0.2564 (3)	0.4542 (2)	0.6385 (2)	0.0078 (3)	
O10	0.2491 (3)	0.0488 (2)	0.3518 (2)	0.0082 (3)	
N1	0.7538 (4)	0.7431 (3)	0.0062 (3)	0.0094 (3)	
H1W	0.360 (8)	0.473 (7)	0.648 (6)	0.082 (19)*	
H2W	0.209 (7)	0.536 (5)	0.628 (5)	0.042 (14)*	
H3W	0.294 (6)	−0.056 (5)	0.364 (4)	0.026 (10)*	
H4W	0.146 (4)	0.046 (4)	0.337 (3)	0.001 (7)*	
H1O	0.724 (6)	0.203 (5)	0.019 (4)	0.053 (12)*	
H1N	0.864 (5)	0.713 (4)	0.051 (4)	0.017 (8)*	
H2N	0.641 (6)	0.712 (4)	0.076 (4)	0.029 (9)*	
H3N	0.782 (5)	0.694 (4)	−0.091 (4)	0.017 (8)*	
H4N	0.730 (9)	0.850 (8)	0.004 (6)	0.10 (2)*	

### catena-Poly[ammonium [[diaquanickel(II)]-µ-(hydrogen sulfato)-µ-sulfato]] (Ni_100K) Atomic displacement parameters (Å^2^)

**Table:**

	*U*^11^	*U*^22^	*U*^33^	*U*^12^	*U*^13^	*U*^23^
Ni1	0.00552 (12)	0.00502 (12)	0.00545 (12)	−0.00102 (9)	−0.00143 (9)	0.00004 (8)
S1	0.0051 (3)	0.0053 (2)	0.0042 (2)	−0.00159 (19)	−0.00140 (19)	0.00091 (17)
S2	0.0044 (3)	0.0058 (2)	0.0046 (2)	−0.00155 (19)	−0.00146 (18)	0.00088 (17)
O1	0.0066 (8)	0.0123 (8)	0.0092 (7)	−0.0039 (6)	−0.0021 (6)	0.0020 (6)
O2	0.0060 (8)	0.0074 (7)	0.0079 (6)	0.0005 (6)	−0.0009 (6)	−0.0023 (5)
O3	0.0098 (8)	0.0045 (7)	0.0076 (6)	−0.0008 (6)	−0.0042 (6)	0.0008 (5)
O4	0.0093 (8)	0.0118 (8)	0.0052 (6)	−0.0045 (6)	−0.0037 (6)	0.0040 (5)
O5	0.0043 (8)	0.0139 (8)	0.0089 (7)	−0.0047 (6)	−0.0034 (6)	0.0024 (6)
O6	0.0121 (9)	0.0051 (7)	0.0083 (6)	−0.0025 (6)	−0.0038 (6)	0.0022 (5)
O7	0.0067 (8)	0.0066 (7)	0.0089 (7)	−0.0007 (6)	−0.0009 (6)	−0.0021 (5)
O8	0.0113 (8)	0.0116 (8)	0.0050 (6)	−0.0059 (6)	−0.0045 (6)	0.0032 (6)
O9	0.0081 (9)	0.0067 (8)	0.0094 (7)	−0.0014 (6)	−0.0035 (6)	−0.0008 (6)
O10	0.0067 (9)	0.0077 (8)	0.0115 (7)	−0.0007 (6)	−0.0050 (6)	−0.0009 (6)
N1	0.0108 (8)	0.0107 (9)	0.0072 (8)	−0.0043 (7)	−0.0013 (7)	−0.0009 (6)

### catena-Poly[ammonium [[diaquanickel(II)]-µ-(hydrogen sulfato)-µ-sulfato]] (Ni_100K) Geometric parameters (Å, º)

**Table:**

Ni1—O2	2.0457 (17)	S2—O7	1.4660 (16)
Ni1—O10	2.0491 (18)	S2—O8	1.5472 (17)
Ni1—O9	2.0513 (18)	O8—H1O	0.88 (4)
Ni1—O3^i^	2.0519 (16)	O9—H1W	0.80 (5)
Ni1—O7	2.0594 (16)	O9—H2W	0.65 (4)
Ni1—O6^ii^	2.0595 (17)	O10—H3W	0.80 (3)
S1—O1	1.4645 (17)	O10—H4W	0.78 (3)
S1—O3	1.4806 (16)	N1—H1N	0.93 (3)
S1—O2	1.4808 (16)	N1—H2N	0.91 (3)
S1—O4	1.4924 (17)	N1—H3N	0.87 (3)
S2—O5	1.4487 (17)	N1—H4N	0.79 (6)
S2—O6	1.4639 (16)		
			
O2—Ni1—O10	87.59 (7)	O5—S2—O7	110.67 (10)
O2—Ni1—O9	92.83 (7)	O6—S2—O7	110.96 (9)
O10—Ni1—O9	179.11 (9)	O5—S2—O8	107.92 (9)
O2—Ni1—O3^i^	89.49 (7)	O6—S2—O8	103.32 (10)
O10—Ni1—O3^i^	86.25 (7)	O7—S2—O8	108.63 (10)
O9—Ni1—O3^i^	94.54 (7)	S1—O2—Ni1	130.45 (10)
O2—Ni1—O7	178.91 (7)	S1—O3—Ni1^i^	130.68 (10)
O10—Ni1—O7	91.91 (7)	S2—O6—Ni1^ii^	131.99 (11)
O9—Ni1—O7	87.68 (7)	S2—O7—Ni1	130.71 (10)
O3^i^—Ni1—O7	89.52 (7)	S2—O8—H1O	117 (3)
O2—Ni1—O6^ii^	91.53 (7)	Ni1—O9—H1W	123 (4)
O10—Ni1—O6^ii^	93.68 (7)	Ni1—O9—H2W	120 (4)
O9—Ni1—O6^ii^	85.52 (7)	H1W—O9—H2W	99 (5)
O3^i^—Ni1—O6^ii^	178.97 (8)	Ni1—O10—H3W	127 (2)
O7—Ni1—O6^ii^	89.46 (7)	Ni1—O10—H4W	117 (2)
O1—S1—O3	111.91 (10)	H3W—O10—H4W	100 (3)
O1—S1—O2	108.25 (10)	H1N—N1—H2N	113 (3)
O3—S1—O2	109.45 (9)	H1N—N1—H3N	111 (3)
O1—S1—O4	109.98 (9)	H2N—N1—H3N	109 (3)
O3—S1—O4	106.50 (10)	H1N—N1—H4N	102 (4)
O2—S1—O4	110.77 (10)	H2N—N1—H4N	106 (4)
O5—S2—O6	114.88 (10)	H3N—N1—H4N	115 (4)

Symmetry codes: (i) −*x*, −*y*+1, −*z*+1; (ii) −*x*+1, −*y*, −*z*+1.

### catena-Poly[ammonium [[diaquanickel(II)]-µ-(hydrogen sulfato)-µ-sulfato]] (Ni_100K) Hydrogen-bond geometry (Å, º)

**Table:**

*D*—H···*A*	*D*—H	H···*A*	*D*···*A*	*D*—H···*A*
O8—H1*O*···O4^iii^	0.88 (4)	1.62 (4)	2.499 (2)	173 (4)
O9—H1*W*···O1^iv^	0.80 (5)	2.08 (5)	2.747 (3)	140 (5)
O9—H2*W*···O2^i^	0.65 (4)	2.55 (4)	3.061 (3)	138 (4)
O9—H2*W*···O5^v^	0.65 (4)	2.69 (4)	3.232 (2)	143 (4)
O9—H1*W*···O7^v^	0.80 (5)	2.44 (5)	2.963 (2)	124 (5)
O10—H3*W*···O1^vi^	0.80 (3)	2.41 (3)	3.115 (2)	148 (3)
O10—H3*W*···O2^vi^	0.80 (3)	2.59 (4)	3.051 (2)	119 (3)
O10—H4*W*···O5^vii^	0.78 (3)	2.00 (3)	2.719 (3)	155 (3)
N1—H3*N*···O3^iii^	0.87 (3)	1.97 (3)	2.834 (3)	172 (3)
N1—H1*N*···O4^v^	0.93 (3)	2.07 (3)	2.980 (3)	169 (3)
N1—H2*N*···O1^i^	0.91 (3)	2.00 (3)	2.903 (3)	174 (3)
N1—H4*N*···O8^viii^	0.79 (6)	2.31 (6)	3.068 (3)	159 (5)

Symmetry codes: (i) −*x*, −*y*+1, −*z*+1; (iii) *x*+1, *y*, *z*−1; (iv) *x*+1, *y*, *z*; (v) −*x*+1, −*y*+1, −*z*+1; (vi) −*x*, −*y*, −*z*+1; (vii) *x*−1, *y*, *z*; (viii) *x*, *y*+1, *z*.
